# Carbon Dioxide Activation at Metal Centers: Evolution of Charge Transfer from Mg ^.+^ to CO_2_ in [MgCO_2_(H_2_O)_*n*_]^.+^, *n=*0–8

**DOI:** 10.1002/anie.202001292

**Published:** 2020-03-12

**Authors:** Erik Barwa, Tobias F. Pascher, Milan Ončák, Christian van der Linde, Martin K. Beyer

**Affiliations:** ^1^ Institut für Ionenphysik und Angewandte Physik Universität Innsbruck Technikerstraße 25 6020 Innsbruck Austria

**Keywords:** ab initio calculations, CO_2_ activation, hydration, IR spectroscopy, mass spectrometry

## Abstract

We investigate activation of carbon dioxide by singly charged hydrated magnesium cations Mg ^.+^(H_2_O)_*n*_, through infrared multiple photon dissociation (IRMPD) spectroscopy combined with quantum chemical calculations. The spectra of [MgCO_2_(H_2_O)_*n*_]^.+^ in the 1250–4000 cm^−1^ region show a sharp transition from *n=*2 to *n=*3 for the position of the CO_2_ antisymmetric stretching mode. This is evidence for the activation of CO_2_ via charge transfer from Mg ^.+^ to CO_2_ for *n*≥3, while smaller clusters feature linear CO_2_ coordinated end‐on to the metal center. Starting with *n=*5, we see a further conformational change, with CO_2_^.−^ coordination to Mg^2+^ gradually shifting from bidentate to monodentate, consistent with preferential hexa‐coordination of Mg^2+^. Our results reveal in detail how hydration promotes CO_2_ activation by charge transfer at metal centers.

## Graphical abstract

**One, two, three**: Three water molecules activate carbon dioxide when coordinated to Mg ^.+^ in the gas phase, pushing electron density from the magnesium center since they are keen on interacting with an Mg^2+^ ion. For the same reason, they squeeze in between CO_2_^.−^ and the metal center if there are five or more. This sequence of electron transfer followed by formation of a solvent‐separated ion pair is unraveled by infrared spectroscopy.

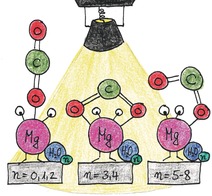

Due to its infrared (IR) active modes,^1^ CO_2_ is the major contribution to the anthropogenic greenhouse effect.^2^ Its chemical inertness limits its use as chemical feedstock,^3^ with CO_2_^.−^ as a key intermediate in many processes.^4^ CO_2_^.−^ in the gas phase is metastable and decays by autodetachment.^5^ However, solvation efficiently stabilizes this radical anion in small clusters.^6^ Gas phase studies on CO_2_ activation have been reviewed recently by Weber5a, 7 and Schwarz.^8^ These cluster experiments serve as a bridge between the gas and condensed phase.^9^ Uggerud, Asmis and co‐workers demonstrated Grignard analogues in the gas phase and identified a bidentate binding motif in the [ClMgCO_2_]^.−^ complex by infrared spectroscopy.^10^ In this case, liberation of CO was observed after reaction with water.10a

Collision‐induced dissociation (CID) experiments and theoretical calculations determined the bond dissociation energies of Mg ^.+^ in small water clusters.^11^ Hydrated singly‐charged magnesium cations undergo an intracluster reaction within a certain size regime forming MgOH(H_2_O)_*n*−1_^+^.^12^ Several reactivity and photochemical studies on Mg ^.+^(H_2_O)_*n*_ confirmed the coexistence of Mg^2+^ and a hydrated electron for *n*>15.^13^ Quantum chemical calculations have corroborated the existence of Mg^2+^/O_2_^.−^ and Mg^2+^/CO_2_^.−^ ion pairs in clusters containing 3 and 16 water molecules.13h, 13i Infrared spectroscopy is an excellent tool for the structural investigation of metal–CO_2_ interactions in clusters such as M^+^(CO_2_)_*n*_ (M=Mg, Al, Si, V, Fe, Co, Ni, Rh, Ir),^14^ or M^−^(CO_2_)_*n*_ (M=Ti, Mn, Fe, Co, Ni, Cu, Ag, Au, Sn, Bi).^15^ A number of IR spectroscopic studies of hydrated ions M^+/−^(H_2_O)_*n*_ have also been performed.^16^ The neutral MgCO_2_ complex in helium nanodroplets has been investigated, showing no evidence for charge transfer.^17^ In addition, IR spectroscopy of the hydrated bicarbonate anion HCO_3_^−^(H_2_O)_1–10_ and the radical anions CO_2_^.−^(H_2_O)_2–61_ and (CO_2_)_*n*_^.−^ has been performed.^18^ Utilizing matrix isolation, two absorptions of CO_2_^.−^ have been observed in a neon matrix.^19^

Here, we investigate CO_2_ activation in [MgCO_2_(H_2_O)_*n*_]^.+^ clusters, *n=*0–8. We probe CO_2_ and CO_2_^.−^ vibrational modes as well as H_2_O bending and stretching modes in the 1250–4000 cm^−1^ region in an FT‐ICR mass spectrometer via infrared multiple photon dissociation (IRMPD) spectroscopy. Quantum chemical calculations provide an interpretation of the measured spectra.

IRMPD spectra of [Mg(CO_2_)(H_2_O)_*n*_]^.+^ with the ICR cell cooled to 80 K are shown in Figure 1 for *n*≤3. For these cluster sizes, fragmentation proceeds by CO_2_ loss, reaction (1).

(1) $$\left[\mathrm{Mg}\left(\mathrm{CO}{}_{2}\right)\left(H{}_{2}O\right){}_{n}\right]{}^{•}{}^{+}+m\cdot h\nu {}_{\mathrm{IR}}\to \;\left[\mathrm{Mg}\left(H{}_{2}O\right){}_{n}\right]{}^{•}{}^{+}+\mathrm{CO}{}_{2},\;\;\;n\leq 3$$

**Figure 1:**
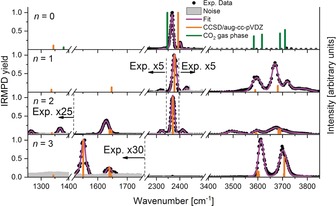
Measured IRMPD spectra of [MgCO_2_(H_2_O)_*n*_]^.+^, *n=*0–3, at *T*≈80 K. Fragmentation proceeds according to reaction (1). IR transitions calculated at the CCSD/aug‐cc‐pVDZ level for **a** (*n=*0–2) and **b** (*n=*3) isomers, respectively, see Figure 2 for structures, are shown as orange bars; scaling of 0.988 was used below 2500 cm^−1^ whereas a factor of 0.95 is used above due to strong anharmonicity of O‐H stretching, see Supporting Information for details. Absorption maxima of a measured CO_2_ spectrum^20^ are added as green bars for comparison.

For the smallest clusters [Mg(CO_2_)(H_2_O)_*n*_]^.+^, *n=*0–2, the IR spectra indicate the presence of a linear, largely unperturbed CO_2_ ligand. The strong absorption at ≈2370 cm^−1^ corresponds to the antisymmetric CO_2_ stretch, *ν*_anti_(C−O), previously observed in [MgCO_2_Ar]^.+^.14b Additionally, weak bands separated by ≈35–55 cm^−1^ above and below this band are recorded. For *n=*0, 1, the branch with lower energy has smaller intensity. These transitions are interpreted to arise due to CO_2_ hindered rotation, *ν*_hr_(CO_2_), calculated to lie at 58 cm^−1^ (*n=*0) within the harmonic approximation. Thus, the higher‐energy branch arises as a combination of *ν*_anti_(C−O) and *ν*_hr_(CO_2_). The lower‐energy branch corresponds to the hot‐band transition, starting with one vibrational quantum in *ν*_hr_(CO_2_). This coupling resembles the situation in HCO_2_^−^(H_2_O).^21^ Further absorptions are observed in the 3450–3800 cm^−1^ region for *n*≥1. Symmetric and antisymmetric O−H stretch vibrations dominate this region, slightly blue‐shifted compared to O−H stretch vibrations in Mg ^.+^(H_2_O)Ar,^22^ with contributions from the well‐known Fermi resonances of CO_2_.^20^

Calculated structures of low‐lying isomers are shown in Figure 2. Several isomer classes were considered: Isomer **a** with a linear CO_2_ for *n*≤4; isomers **b** and **c** with an activated CO_2_ possessing a bidentate motif *η* ^2^‐O and a monodentate motif *η* ^1^‐O, respectively; isomer **d** featuring a solvent‐separated Mg^2+^/CO_2_^.−^ ion pair for *n*≥5.13h As long as the unpaired electron stays on magnesium, asymmetric solvation is preferred, similar to Mg ^.+^(H_2_O)_*n*_.12b IR transitions calculated at the CCSD/aug‐cc‐pVDZ level in harmonic approximation for structures **0 a**, **Ia** and **IIa** reproduce the main features of the measured spectra, Figure 1. The position of CO_2_ vibrations after complexation to the Mg ^.+^(H_2_O)_*n*_ cation, *n=*0–2, differs only negligibly; thus, CO_2_ stays almost uninfluenced by the Mg ^.+^(H_2_O)_*n*_ cation. In fact, the IR spectrum of [Mg(CO_2_)(H_2_O)_2_]^.+^ resembles closely a linear combination of CO_2_ and Mg ^.+^(H_2_O)_2_ IR spectra (see Figure S4 in the Supporting Information). Accordingly, calculations also predict no electron transfer to CO_2_ for those isomers, with the overall positive charge shared by the carbon dioxide molecule (Figure 2).

**Figure 2:**
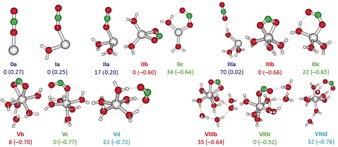
Calculated isomers of [MgCO_2_(H_2_O)_*n*_]^.+^ for *n=*0–3, 5 and 8 at the M06L/aug‐cc‐pVDZ level of theory. Relative energies are given in kJ mol^−1^. The charge on the CO_2_ unit (in *e*) is given in brackets. See Figure S3 for structures with *n=*4, 6, 7.

While for *n=*0, 1, the laser system is not powerful enough for IRMPD below 1800 cm^−1^, we succeeded to measure the IR spectrum also in the 1250–1800 cm^−1^ region for *n=*2. In the symmetric C−O stretch region, we see bands at 1265 and 1369 cm^−1^. The calculations predict a splitting of the degenerate CO_2_ bending mode due to the interaction with Mg ^.+^ and water in isomer **IIa** at 654 cm^−1^ and 658 cm^−1^, along with the fundamental symmetric C−O stretch at 1339 cm^−1^. The discrepancy arises due to a Fermi resonance between the overtone/combination band of the CO_2_ bending with the symmetric C−O stretch vibration within the linear CO_2_ molecule. This Fermi interaction is well documented in the literature for CO_2_, shifting the corresponding two vibrations to about 1285 and 1390 cm^−1^, respectively.^23^ Complexation with Mg ^.+^ shifts these bands slightly to the red. The remaining band at 1628 cm^−1^ is assigned as water bending mode, in good agreement with calculations (Figure 1).

For *n=*2, the isomer with activated CO_2_, **IIb**, is already more stable by 17 kJ mol^−1^ compared to **IIa**. However, the activation of CO_2_ via electron transfer from a doubly hydrated Mg ^.+^ center, starting from isomer **IIa,** faces a substantial barrier of 41 kJ mol^−1^, Figure S5, compared to a CO_2_ dissociation energy of 38 kJ mol^−1^. Thus, isomers **IIb** and **IIc** are not formed in the experiment, as the entropically favored dissociation prevails over CO_2_ activation, and only isomer **IIa** is observed. Isomers with activated CO_2_, **IIb**/**IIc**, also cannot arise from evaporation of a water molecule from the next heavier cluster, [Mg(CO_2_)(H_2_O)_3_]^.+^, as CO_2_ loss is preferred for *n*≤3, see Table S1, in agreement with experiment.

In the IR spectrum of *n=*3, we observe a fundamental change: the antisymmetric stretch of CO_2_ at ≈2370 cm^−1^ shifts to 1552 cm^−1^. This is clear evidence of CO_2_ activation via electron transfer from Mg ^.+^ to CO_2_, forming an Mg^2+^⋅⋅⋅CO_2_^.−^ ion pair. This electron transfer is driven by the higher water binding energy of Mg^2+^ compared to Mg ^.+^.11b The bidentate binding motif **IIIb** is energetically slightly preferred over the monodentate **IIIc,** while isomer **IIIa** containing linear CO_2_ is 70 kJ mol^−1^ less stable than **IIIb**.

To further probe the influence of the hydration shell on the Mg^2+^/CO_2_^.−^ ion pair, we recorded IRMPD spectra of [Mg(CO_2_)(H_2_O)_*n*_]^.+^ up to *n=*8 in the 1250–1800 cm^−1^ region at 80 K, shown in Figures 3 and S1. Figure S2 provides spectra at room temperature for comparison. In agreement with the calculated thermochemistry, H_2_O evaporates exclusively for *n*≥4, reaction (2).

(2) $$\left[\mathrm{Mg}\left(\mathrm{CO}{}_{2}\right)\left(H{}_{2}O\right){}_{n}\right]{}^{•}{}^{+}+m\cdot h\nu {}_{\mathrm{IR}}\to \;\left[\mathrm{Mg}\left(\mathrm{CO}{}_{2}\right)\left(H{}_{2}O\right){}_{n-1}\right]{}^{•}{}^{+}+H{}_{2}O,\;n\geq 4$$

**Figure 3:**
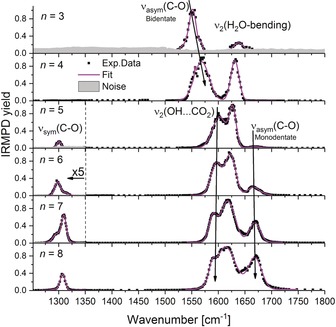
Measured IRMPD spectra of [MgCO_2_(H_2_O)_*n*_]^.+^ for *n=*3–8 at *T* ≈80 K. See the text and reactions (1) and (2) for fragmentation channels.

For *n=*3, 4, we observe a band at 1552 cm^−1^ that can be assigned as the antisymmetric stretch of CO_2_^.−^, *ν*_anti_(C−O), in isomer **IIIb** with bidentate bonding motif.18b The whole absorption band at 1600–1650 cm^−1^ arises due to water vibrations. Unfortunately, the laser power is not sufficient to observe fragmentation in the *ν*_sym_(C−O) symmetric stretching region of the CO_2_^.−^ anion in the cooled cell, for *n=*3 not even at room temperature (Figure S2). This transition is calculated at the M06L/aug‐cc‐pVDZ levels of theory to be at 1317 and 1259 cm^−1^ for isomers **IIIb** and **IIIc**, respectively. However, isomer **IIIc** should exhibit a very intense absorption at 2863 cm^−1^, which corresponds to the OH stretch directed to CO_2_^.−^. Due to the lack of this band in the experiment, we conclude to have only isomer **IIIb**, that is, CO_2_^.−^ is attached to Mg^2+^ in bidentate manner. Analogously, the structure observed for *n=*4 is **IVb**; however, the monodentate structure **IVc** is also observed for *n=*4 at room temperature when more energy is available (Figure S2), evidenced by the additional band at 1678 cm^−1^. Charge analysis shows that charge transfer takes place from Mg ^.+^, with partial charges on the CO_2_ moiety from −0.6 e to −0.7 e.

For *n*>4, interpretation of the band structure in the 1550–1750 cm^−1^ region is getting more complicated, since the bidentate, monodentate and solvent separated isomers get closer in energy. In addition, the vibrational frequencies predicted by our quantum chemical calculations strongly depend on the functional, while the relative energies of the isomers are quite robust (see the Supporting Information). We therefore rely on the interpretation of the experimental spectrum based on thermochemical arguments and qualitative trends.

The feature at about 1670 cm^−1^, indicative of the monodentate structure, emerges for *n=*5 and becomes more prominent with increasing cluster size. At the same time, the band at ≈1560 cm^−1^ is severely weakened, and a new band at 1600 cm^−1^ appears, which we assign to the H_2_O bending mode, *ν*_2_(O−H⋅⋅⋅CO_2_), involving an interaction of OH groups with the monodentate CO_2_^.−^ ligand. The gradual switching between bidentate and monodentate structures from *n=*4 to *n=*5 can be rationalized by the preferred hexacoordination of Mg^2+^, in line with calculated thermochemistry shown in Figures 2 and S3.

A new band appears at about 1300 cm^−1^ for *n*>4, which we assign to *ν*_sym_(C−O) of the monodentate isomers. The structure of the band indicates the presence of several isomers. Interestingly, the change from bidentate to monodentate binding motif does not affect the partial charge of the CO_2_^.−^ ligand. With increasing number of water molecules, the water bending region gets more and more congested, reflecting the number of distinguishable H_2_O molecules in the cluster and the increasing number of energetically accessible isomers. It is plausible that also solvent‐separated isomers contribute to the spectrum, however without a clear spectral assignment. Earlier calculations indicated that solvent separated ion pair and monodentate contact ion pair structures lie within 10 kJ mol^−1^ for *n=*16, with the solvent separated structure slightly preferred.13h

The measured IR spectra of [Mg(CO_2_)(H_2_O)_*n*_]^.+^ clusters show a clear dependence of CO_2_ activation on the number of water molecules. For *n=*2, although CO_2_ activation is thermochemically preferred, it is hindered by a barrier. For *n*>2, we observe charge transfer from Mg ^.+^ to CO_2_, with the resulting CO_2_^.−^ coordinated to Mg^2+^ initially in bidentate fashion. With increasing cluster size, monodentate coordination and solvent separated ion pair structures take over. Our results emphasize the role of water in the activation of CO_2_ on metal centers.

## Experimental Section

The experiments are performed on a modified 4.7 T FT‐ICR Bruker/Spektrospin CMS47X mass spectrometer equipped with an external laser vaporization source.^24^ A pulsed frequency doubled Nd:YAG laser is focused onto a rotating isotopically enriched magnesium target (^24^Mg, 99.9 %). The resulting plasma is entrained into a gas pulse of He, H_2_O and CO_2_, undergoing supersonic jet expansion. The temperature of the cylindrical cell is lowered for most experiments to *T* ≈80 K via liquid nitrogen cooling^25^ to reduce the contribution of black body infrared radiative dissociation (BIRD).^26^ Radiation from tunable OPO laser systems (EKSPLA NT273‐XIR, EKSPLA NT277) is coupled into the ICR cell. For evaluation we use the IRMPD yield.^27^ We defined this previously^28^ as ∑(photofragments)/∑(precursor+photofragments)/*P*/*t*, where *P* the laser power measured directly after the experiment and *t* the irradiation time, with each spectrum normalized to the maximum value. Further details on the experimental setup are found in the Supporting Information.

The structure and properties of [Mg(CO_2_)(H_2_O)_*n*_]^.+^ clusters (*n=*0–8) were studied employing CCSD/aug‐cc‐pVDZ and M06L/aug‐cc‐pVDZ theory levels, see Supporting Information for benchmarking calculations (Tables S2–S5). While thermochemical values at the CCSD level are reproduced well by selected DFT functionals, vibrational frequencies have a relatively large error, with M06L providing the lowest one on average. Therefore, M06L is used for frequency calculations of larger clusters. Vibrational spectra are scaled by a factor of 0.988 and 0.97 for CCSD and M06L calculations, respectively. A factor of 0.95 is used for CCSD calculations above 2500 cm^−1^ due to the high anharmonicity of O‐H stretching vibrations in hydrated metal cations.16k Wavefunction stabilization was performed, all considered structures represent local minima. Partial charges were calculated within the CHELPG Scheme.^29^ The Gaussian 16 software was employed.^30^

## Conflict of interest

The authors declare no conflict of interest.

## Supporting information

As a service to our authors and readers, this journal provides supporting information supplied by the authors. Such materials are peer reviewed and may be re‐organized for online delivery, but are not copy‐edited or typeset. Technical support issues arising from supporting information (other than missing files) should be addressed to the authors.

Supplementary
