# Supplementary material for: Carbon Dioxide Activation at Metal Centers: Evolution of Charge Transfer from Mg .+ to CO2 in [MgCO2(H2O)n].+, n=0–8
Source: Angew Chem Int Ed Engl. 2020 Mar 12;59(19):7467–71. doi: 10.1002/anie.202001292 (PMC7217156; doi:10.1002/anie.202001292)
Supplement: Supplementary file 1 — Supplementary [file ANIE-59-7467-s001.pdf]

Angewandte Chemie
Eine Zeitschrift der Gesellschaft Deutscher Chemiker

Supporting Information

Carbon Dioxide Activation at Metal Centers: Evolution of Charge Transfer from $Mg^{\cdot +}$ to CO_2 in $[MgCO_2(H_2O)_n]^{\cdot +}$, $n = 0–8$

Erik Barwa+, Tobias F. Pascher+, Milan Ončák, Christian van der Linde, and Martin K. Beyer**

anie_202001292_sm_miscellaneous_information.pdf

Energetics of dissociation channels

Table S1. Dissociation energies of $[MgCO_2(H_2O)_n]^{\bullet+}$ for loss of H_2O and CO_2, respectively. Calculated at the M06L/aug-cc-pVDZ level of theory. All energies are given in kJ/mol relative to the most stable isomer.

n	0	1	2	3	4	5	6	7	8
H_2O loss	-	114	111	124	91	72	67	59	47
CO_2 loss	64	49	55	90	116	126	125	135	143

Experimental Setup

Either a Continuum Surelite II (10 Hz) or a Litron Nano S60-30 (30 Hz) is used as vaporization laser for an isotopically enriched ^{24}Mg (99.9%) target. The pick-up gas consisting of helium seeded with CO_2 is supersonically expanded, passed through a skimmer and then through a set of electrostatic lenses which guide the ions through differential pumping stages[1] into the ICR infinity cell.[2] In the ICR cell, the ions are trapped in an electromagnetic field under ultra-high vacuum conditions (~10^{-10} mbar) in the center of a 4.7 T superconducting magnet as explained in detail by Marshall et al.[3] The ions are then resonantly excited and their cyclotron frequency is measured.[3] For each data point, 20 to 50 spectra are accumulated and averaged to obtain a higher signal-to-noise ratio.

For spectroscopy, ions are mass selected and irradiated by infrared (IR) radiation from a 1000 Hz diode pumped EKSPLA NT273-XIR or EKSPLA NT277 laser system. Each data point in the absorption spectra corresponds to a full mass spectrum, measured after irradiation with a preset irradiation time (0.6–20 s). The IR/OPO laser system EKSPLA NT273-XIR operates between 4476 and 12000 nm whereas the EKSPLA NT277 operates from 2500 to 4475 nm. The measurements for $n \geq 4$ were recorded at 1250–2234 cm^{-1}. Because the antisymmetric stretching mode of linear CO_2 lies above 2234 cm^{-1}, measurements were recorded at 1250–

4000 cm^{-1} for $n = 0–3$. Immediately after each mass spectrum, the laser power is measured before the laser is tuned to the next wavelength. The wavelength is calibrated using a HighFinesse Laser Spectrum Analyzer IR-III. For the correction of the photon loss by the CaF_2 window, the transmission curve provided by ThorLabs was used.[4] Photodissociation of the complex can occur *via* vibrational resonant excitation during laser irradiation and/or *via* BIRD. To account properly for the influence of BIRD, every tenth to thirtieth measurement is performed without irradiation to gain information on the relative abundance of the BIRD fragments and the precursor ion. These fragment ion signals due to BIRD I_{BIRD} are subtracted from the mass spectra with irradiation of the laser I_0: $I_{korr} = I_0 - I_{BIRD}$.

For larger clusters, BIRD has a stronger influence. At room temperature, after a trapping time of 1 s, roughly 75% of the $MgCO_2(H_2O)_8^+$ cluster loose one water molecule, forming $Mg(CO_2)(H_2O)_7^+$. To yield the corrected IR spectrum of $n = 7$ at room temperature, the cluster with $n = 8$ water molecules was isolated to maximize $n = 7$ as precursor. Then the remaining depletion of $n = 8$ (due to radiation) is added to the relative abundance of the $n = 7$ precursor ion.

Benchmarking of Quantum Chemical Calculations

In Table S2, we present benchmarking of methods and basis sets for the decarboxylation and water evaporation reactions of $Mg(H_2O)_2CO_2^+$ and $Mg(H_2O)_3CO_2^+$ along with the comparison of the three different binding motifs of the CO_2 ligand. Additionally, a direct comparison of methods and basis sets to CCSD is shown in Table S3. The aug-cc-pVDZ basis set yields values close to the ones at the triple zeta basis set level. Comparing the methods against CCSD, all DFT-based methods and MP2 yield acceptable values while the best agreement is reached for BMK, M06 and M06L.

Next, we benchmarked the infrared spectra obtained for $Mg(H_2O)_2CO_2^+$ and $Mg(H_2O)_3CO_2^+$ in Tables S4 and S5, respectively, considering three possible binding motifs of the CO_2 molecule to Mg^+. The basis set does not influence results considerably; all methods reproduce the vibrations calculated at the CCSD level apart from few exceptions with errors over 100 cm^{-1}. Based on these results, M06L was chosen due to its lowest average discrepancy compared to CCSD.

Table S2: Energy benchmarking of methods and basis sets for several reactions and three different binding motives of the CO_2 molecule on the Mg center (bidentate, monodentate, linear). Energies are corrected for zero point energy and given in kJ/mol.

reactant	product(s)	B3LYP/aug-cc-pVTZ	B3LYP/aug-cc-pVDZ	BMK/aug-cc-pVDZ	M06/aug-cc-pVDZ	M06L/aug-cc-pVDZ	MP2/aug-cc-pVDZ	CCSD/aug-cc-pVDZ
$Mg(H_2O)_2CO_2^+$	$Mg(H_2O)_2^+ + CO_2$	36	37	42	48	55	28	52
$Mg(H_2O)_2CO_2^+$	$Mg(H_2O)CO_2^+ + H_2O$	93	94	101	104	111	83	105
$Mg(H_2O)_3CO_2^+$	$Mg(H_2O)_3^+ + CO_2$	67	69	85	85	90	67	89
$Mg(H_2O)_3CO_2^+$	$Mg(H_2O)_2CO_2^+ + H_2O$	112	113	123	126	124	123	122
$Mg(H_2O)_2CO_2^+$ Bidentate	Monodentate	27	26	35	30	34	35	37
$Mg(H_2O)_2CO_2^+$ Bidentate	Linear	9	9	16	12	17	-5	17
$Mg(H_2O)_3CO_2^+$ Bidentate	Monodentate	9	9	18	18	22	19	21

$Mg(H_2O)_3CO_2^+$ Bidentate	Linear	48	49	68	65	70	43	65

Table S3: Absolute difference |Δ(Basis)| between aug-cc-pVTZ and aug-cc-pVDZ basis sets for the B3LYP method for reactions shown in Table S2 along with its average, in kJ/mol; difference between the predicted energy of various methods (B3LYP, BMK, M06, M06L, MP2) and the CCSD method using aug-cc-pVDZ, in kJ/mol.

		|Δ(Basis)|	|Δ(Method-CCSD)| using aug-cc-pVDZ				
reactant	product(s)	B3LYP	B3LYP	BMK	M06	M06L	MP2
$Mg(H_2O)_2CO_2^+$	$Mg(H_2O)_2^+ + CO_2$	1	15	9	3	3	23
$Mg(H_2O)_2CO_2^+$	$Mg(H_2O)CO_2^+ + H_2O$	1	11	4	1	6	22
$Mg(H_2O)_3CO_2^+$	$Mg(H_2O)_3^+ + CO_2$	1	20	4	4	1	22
$Mg(H_2O)_3CO_2^+$	$Mg(H_2O)_2CO_2^+ + H_2O$	0	9	1	4	2	1
$Mg(H_2O)_2CO_2^+$ Bidentate	Monodentate	1	11	2	7	3	2
$Mg(H_2O)_2CO_2^+$ Bidentate	Linear	1	8	1	5	1	22
$Mg(H_2O)_3CO_2^+$ Bidentate	Monodentate	0	12	3	4	1	2
$Mg(H_2O)_3CO_2^+$ Bidentate	Linear	1	16	2	0	5	22
Average		**1**	**13**	**3**	**4**	**3**	**15**

Table S4: Benchmarking of IR frequency *v* (cm^{-1}) and intensity *I* (km/mol) in $Mg(H_2O)_2CO_2^+$ using several methods (B3LYP, BMK, M06, M06L, MP2) against CCSD calculations using aug-cc-pVDZ along with a basis set comparison |Δ(Basis)| of aug-cc-pVTZ against aug-cc-pVDZ using B3LYP. Three isomers (monodentate bound CO_2, bidentate bound CO_2 and linear CO_2) are considered. Maxima $|\Delta i|_{max}$ and average values |Δ*i*| are shown for each column. Calculations are not scaled.

CCSD/aug-cc-pVDZ	|Δ(Method-CCSD)| using aug-cc-pVDZ					|Δ(Basis)|
	B3LYP	BMK	M06	M06L	MP2	

	v	I	$\|\Delta v\|$	$\|\Delta I\|$	$\|\Delta v\|$	$\|\Delta I\|$	$\|\Delta v\|$	$\|\Delta I\|$	$\|\Delta v\|$	$\|\Delta I\|$	$\|\Delta v\|$	$\|\Delta I\|$	$\|\Delta v\|$	$\|\Delta I\|$
$Mg(H_2O)_2CO_2^+$ Bidentate	1350	25	21	22	66	6	19	19	1	21	17	2	11	0
	1559	632	14	68	88	113	86	215	50	71	11	24	8	5
	1673	181	33	6	9	113	57	185	6	12	23	6	22	14
	1675	51	34	3	19	5	53	6	4	5	23	1	23	1
	3781	225	41	1	57	53	1	50	6	9	31	20	3	1
	3783	18	41	2	58	5	2	4	6	12	31	5	3	0
	3875	169	50	18	48	50	4	22	5	25	17	16	12	19
	3875	242	50	5	48	51	4	74	5	26	17	28	12	11
$Mg(H_2O)_2CO_2^+$ Monodentate	1257	157	25	122	61	58	31	109	18	107	3	5	9	5
	1596	506	45	25	7	52	38	109	6	46	33	56	21	9
	1680	125	35	3	20	16	54	16	8	13	23	0	22	7
	1731	323	3	68	68	114	51	161	19	41	20	21	6	12
	3172	842	294	16	79	105	67	54	211	85	224	255	14	22
	3775	144	45	20	51	30	2	11	7	23	31	17	4	2
	3862	221	54	4	44	45	2	34	8	5	18	22	12	4
	3891	211	60	47	44	25	11	2	16	48	24	29	8	2
$Mg(H_2O)_2CO_2^+$ Linear	1355	51	6	29	60	21	28	30	10	35	46	9	13	2
	1660	142	40	13	19	8	57	13	3	33	24	6	19	18
	1667	129	38	7	19	19	53	6	11	12	24	1	17	1
	2399	1078	5	141	104	29	78	135	72	204	5	215	11	24
	3780	60	62	52	63	23	6	34	10	36	30	11	5	1
	3784	43	60	22	61	2	3	6	11	6	28	14	4	2
	3877	151	61	1	57	34	7	36	5	15	16	18	12	7
	3885	150	68	3	53	34	1	47	7	24	13	17	13	4
Average $\|\Delta i\|$			**49**	**29**	**50**	**42**	**30**	**57**	**21**	**38**	**30**	**33**	**12**	**7**
$\|\Delta i\|_{max}$			**294**	**141**	**104**	**114**	**86**	**215**	**211**	**204**	**224**	**255**	**23**	**24**

Table S5: Benchmarking of IR frequency *ν* (cm^{-1}) and intensity *I* (km/mol) of $Mg(H_2O)_3CO_2^+$ using several methods (B3LYP, BMK, M06, M06L, MP2) against CCSD calculations using aug-cc-pVDZ along with a basis set comparison |Δ(Basis)| of aug-cc-pVTZ against aug-cc-pVDZ using B3LYP. Three isomers (monodentate bound CO_2, bidentate bound CO_2 and linear CO_2) are considered. Maxima $|\Delta i|_{max}$ and average values |Δ*i*| are shown for each column. Calculations are not scaled.

	CCSD/aug-cc-pVDZ		|Δ(Method-CCSD)| using aug-cc-pVDZ										|Δ(Basis)|	
			B3LYP		BMK		M06		M06L		MP2			
	ν	*I*	|Δ*ν*|	|Δ*I*|	|Δ*ν*|	|Δ*I*|	|Δ*ν*|	|Δ*I*|	|Δ*ν*|	|Δ*I*|	|Δ*ν*|	|Δ*I*|	|Δ*ν*|	|Δ*I*|
$Mg(H_2O)_3CO_2^+$ Bidentate	1361	38	15	22	65	3	20	21	3	26	18	5	10	1
	1570	738	9	11	58	173	31	297	47	69	10	6	8	4
	1660	202	35	0	22	20	54	9	6	1	24	0	23	11
	1664	82	33	26	17	185	3	251	9	22	22	21	18	14
	1668	24	34	15	23	8	53	27	7	12	23	14	21	4
	3788	130	39	3	53	33	2	34	4	5	31	16	1	1
	3793	109	39	4	57	14	4	17	4	5	32	12	1	5
	3795	49	38	4	58	35	5	21	4	7	32	5	1	2
	3900	68	48	23	53	57	6	58	5	25	18	7	11	10
	3901	54	48	3	55	27	8	17	4	4	18	7	12	4
	3902	396	48	19	55	43	9	54	4	38	18	52	11	21
$Mg(H_2O)_3CO_2^+$ Monodentate	1291	174	9	103	66	43	24	112	7	116	3	22	9	8
	1618	556	44	52	3	105	41	171	1	102	30	28	22	16
	1665	128	32	8	21	4	54	6	5	0	24	1	22	8
	1676	113	34	3	17	17	53	15	8	7	24	2	22	3
	1723	268	8	66	66	155	49	202	22	64	17	2	7	23
	3096	1014	289	101	81	104	27	88	145	8	196	212	1	12
	3786	126	37	5	59	33	4	26	4	3	31	8	2	7
	3794	91	37	9	63	24	9	19	0	2	30	13	3	5
	3882	188	45	10	55	50	11	50	0	8	15	18	12	5
	3892	182	50	1	51	47	5	30	5	15	18	8	12	5
	3901	182	51	35	59	23	3	20	10	28	24	57	9	3

$Mg(H_2O)_3CO_2^+$ Linear	1367	19	3	4	62	4	31	4	15	6	50	2	14	1
	1658	128	41	10	22	40	60	4	7	21	24	12	18	15
	1660	129	41	22	22	8	57	14	6	13	24	4	19	4
	1672	101	38	1	22	19	53	10	3	9	25	4	21	1
	2396	1015	12	94	112	27	86	84	82	209	1	215	11	38
	3726	524	82	66	36	133	37	158	43	104	50	83	11	18
	3777	24	63	4	68	29	2	6	7	1	29	9	4	2
	3777	69	63	52	70	14	1	46	7	17	28	15	4	4
	3855	219	65	28	61	24	0	2	11	18	19	11	9	23
	3877	133	65	7	63	36	7	37	1	5	13	13	13	1
	3878	145	64	10	65	23	9	35	2	37	13	19	12	5
Average $\|\Delta i\|$			**47**	**25**	**50**	**47**	**25**	**59**	**15**	**30**	**28**	**27**	**11**	**9**
$\|\Delta i\|_{max}$			**289**	**103**	**112**	**185**	**86**	**297**	**145**	**209**	**196**	**215**	**23**	**38**

The scaling factor for theoretical calculations for CCSD is chosen to fit for $n = 1$ within the C-O fingerprint region, resulting in a factor of 0.988. The observed O-H stretching modes of the $MgCO_2(H_2O)_n^+$ $n = 1$–3 yielded values well below the ones predicted by CCSD calculations. Anharmonic calculations at the MP2/aug-cc-pVTZ level of theory predict a shift of 172 and 199 cm^{-1} in the case of $Mg(CO_2)(H_2O)^+$ for the symmetric and antisymmetric OH stretch vibration, respectively. This high anharmonicity of the O-H stretching mode in hydrated metal cations is well documented in the literature.[5] To correct this, a scaling factor of 0.988 and 0.95 for CCSD calculations is used below and above 2500 cm^{-1}, respectively. For M06L a scaling factor of 0.97 was chosen as best fit overall.

Further Experimental and Computational Data

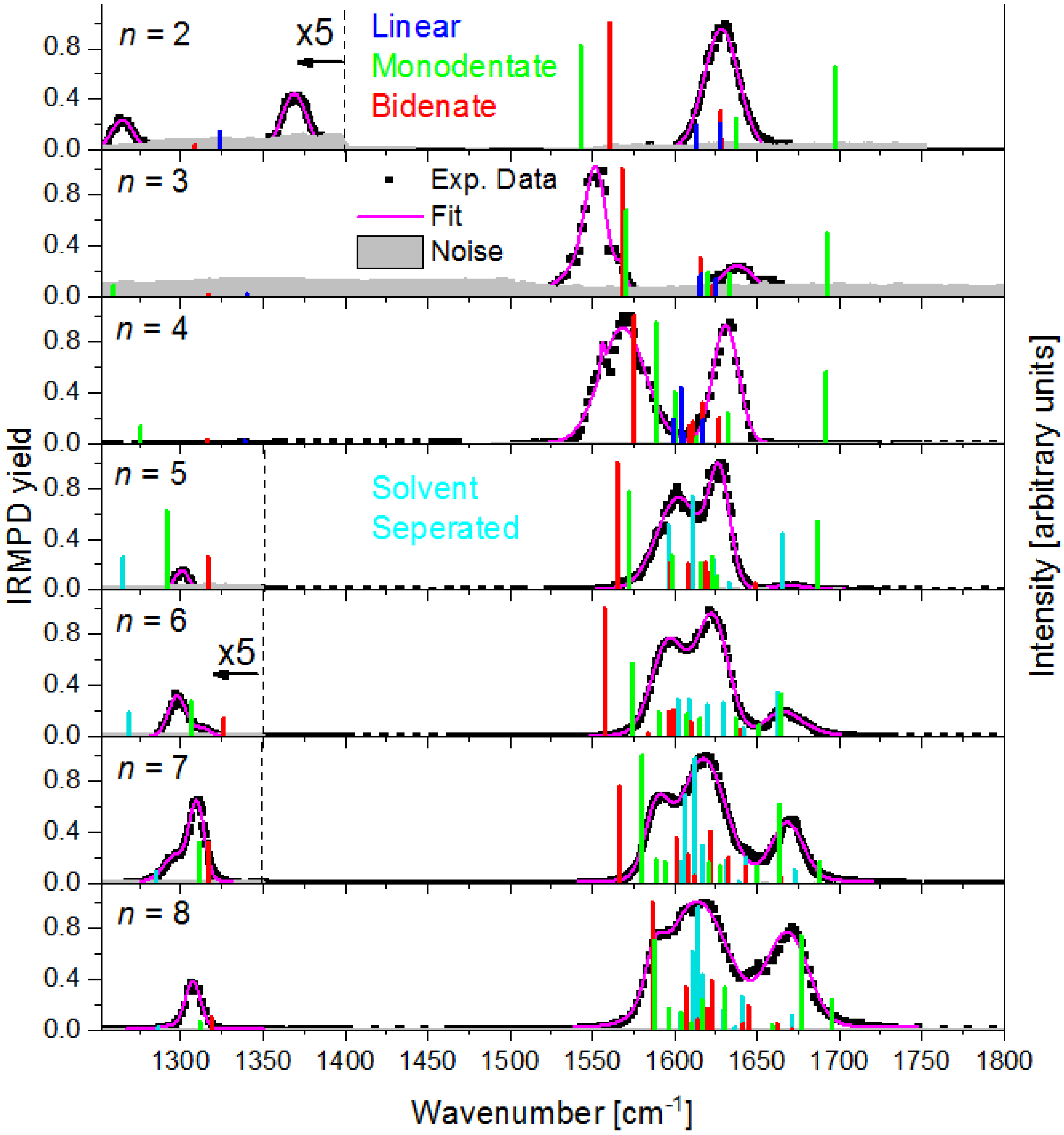

Figure S1: Measured IRMPD spectra of $MgCO_2(H_2O)_n^+$ for $n = 2–8$ at T ≈ 80 K. The bars correspond to transitions of isomers seen in Figure SI3 calculated at the M06L/aug-cc-pVDZ level of theory scaled by a factor of 0.97.

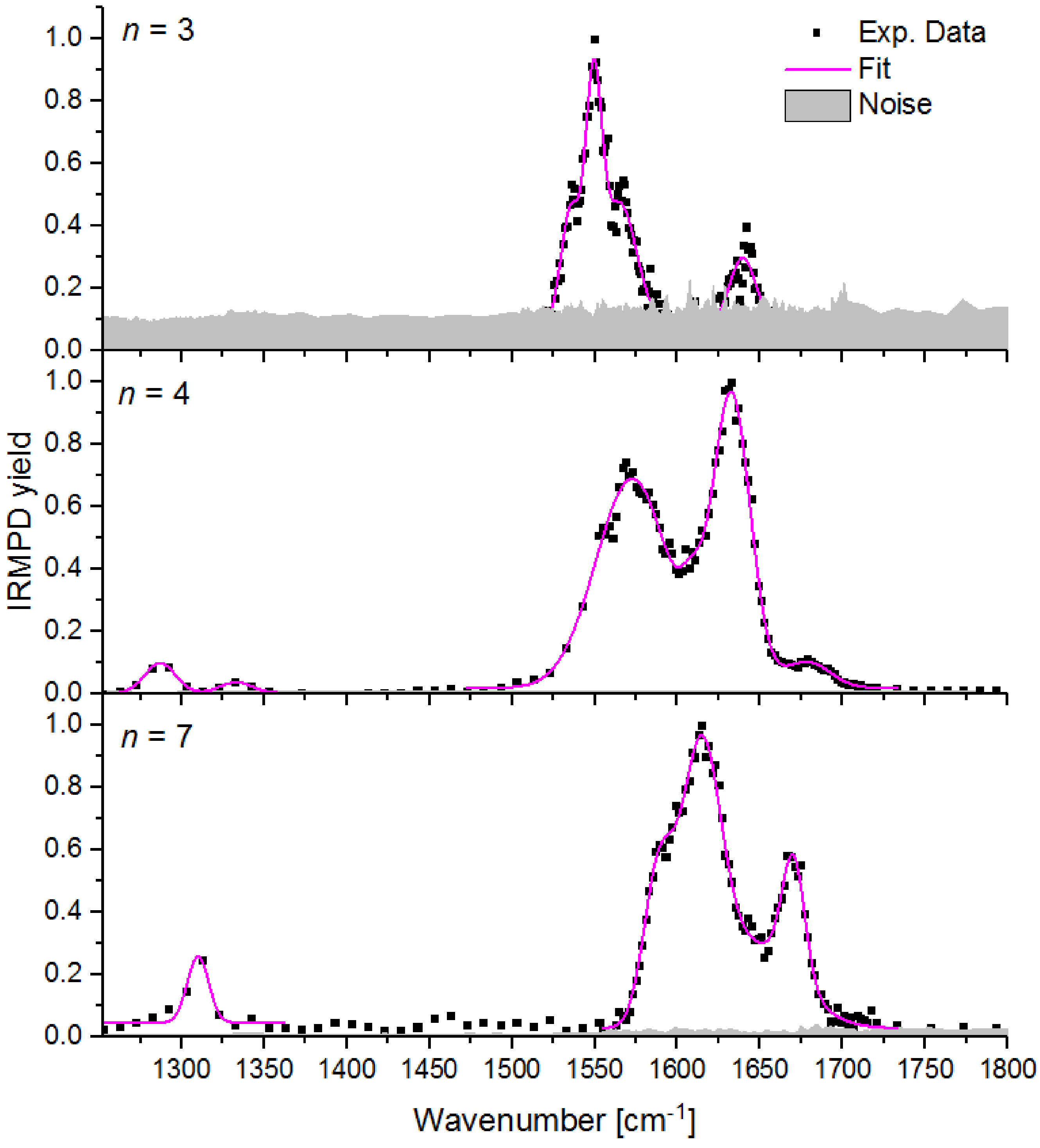

Figure S2: Measured IRMPD spectra of $MgCO_2(H_2O)_n^+$ for *n* = 3, 4 and 7 at room temperature.

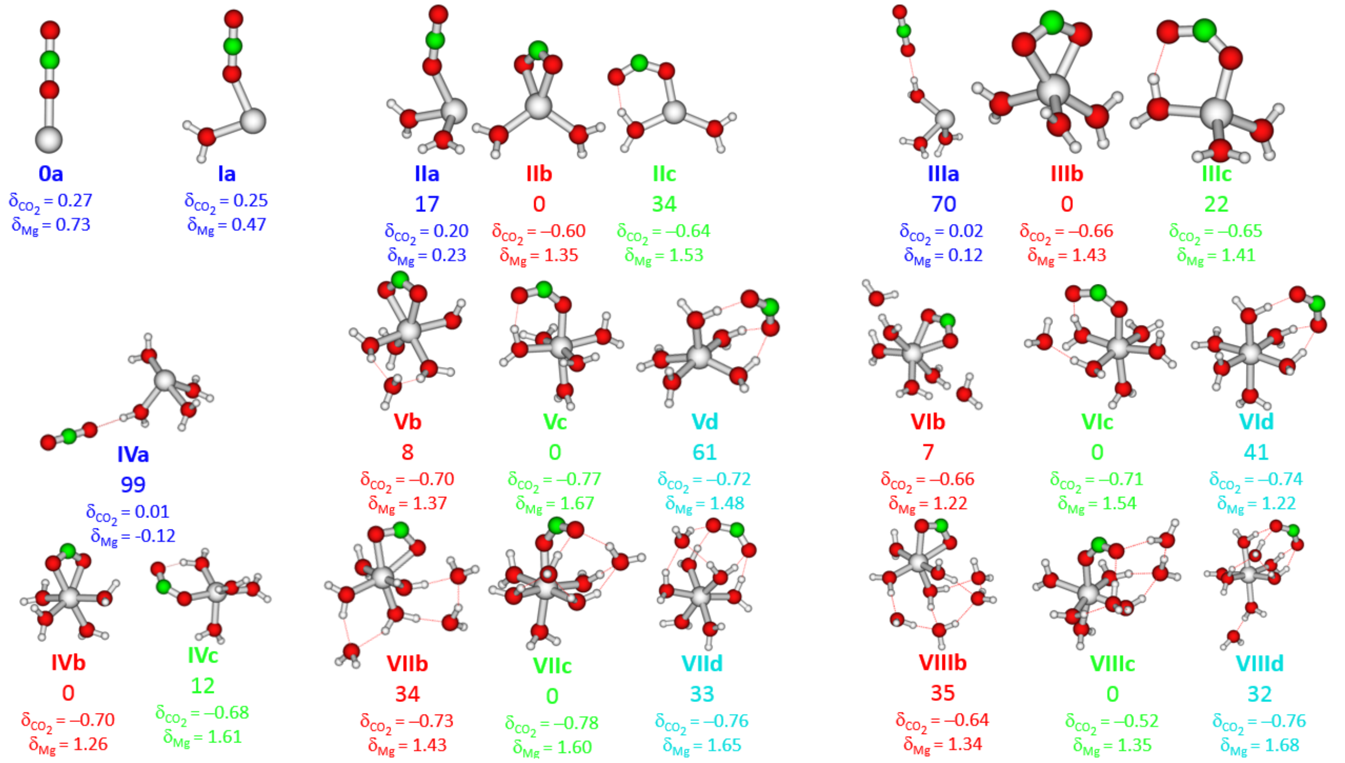

Figure S3: Calculated structures of $MgCO_2(H_2O)_n^+$ on the M06L/aug-cc-pVDZ level of theory along with charges calculated using the CHELPG scheme.

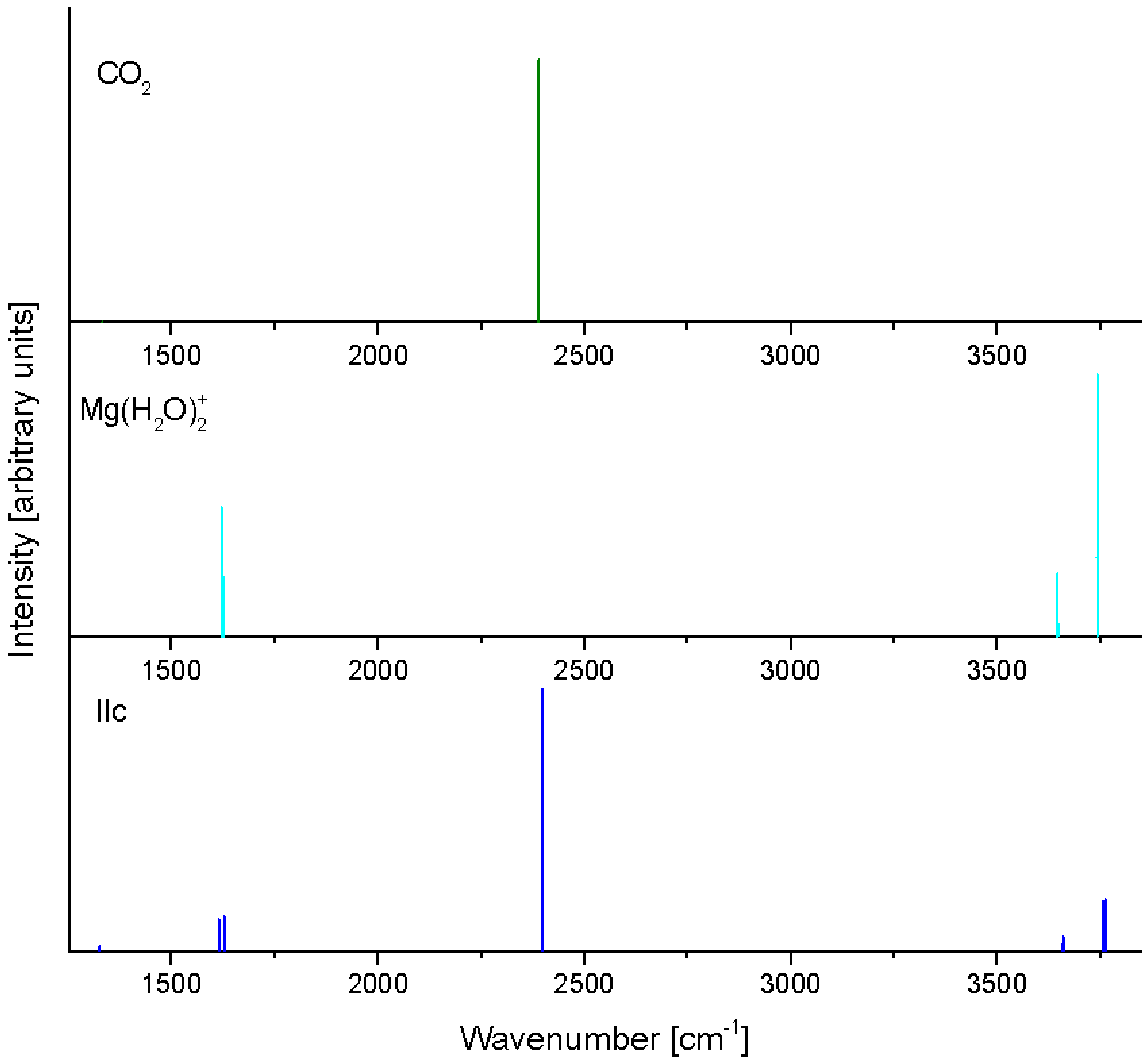

Figure S4: Comparison of IR absorption spectra of **IIa**, $Mg(H_2O)_2^+$ and CO_2 calculated on the M06L/aug-cc-pVDZ level of theory scaled by a factor of 0.97.

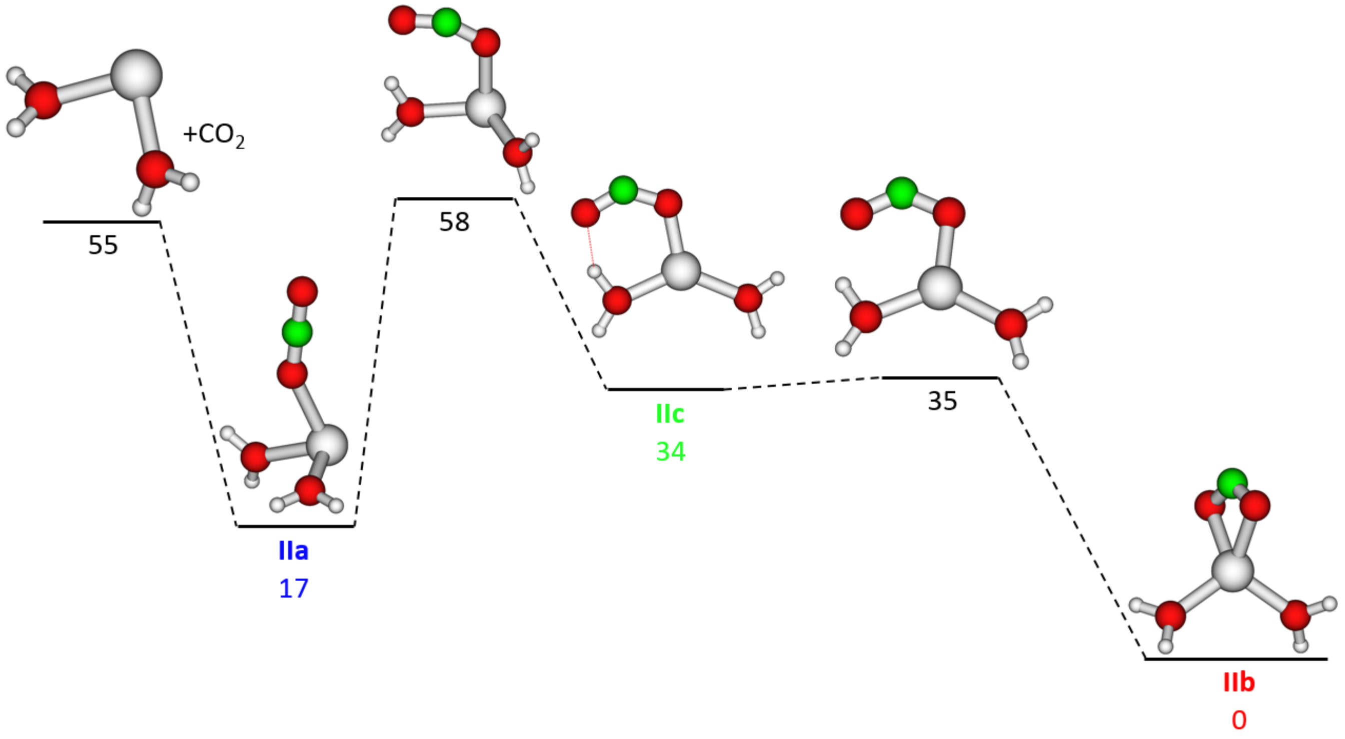

Figure S5: Potential energy surface (energy in kJ/mol) of $MgCO_2(H_2O)_2^+$ calculated on the M06L/aug-cc-pVDZ level of theory.

References

1 M. Beyer, C. Berg, H. W. Görlitzer, T. Schindler, U. Achatz, G. Albert, G. Niedner-Schatteburg and V. E. Bondybey, *J. Am. Chem. Soc.*, 1996, **118**, 7386–7389.

2 P. Caravatti and M. Allemann, *Org. Mass Spectrom.*, 1991, **26**, 514–518.

3 A. G. Marshall, C. L. Hendrickson and G. S. Jackson, *Mass Spectrom. Rev.*, 1998, **17**, 1–35.

4 ThorLabs, *Calcium Fluoride Windows*. September 2019, available at: https://www.thorlabs.com/newgrouppage9.cfm?objectgroup_id=3978.

5 B. Bandyopadhyay, K. N. Reishus and M. A. Duncan, *J. Phys. Chem. A*, 2013, **117**, 7794–7803.

Cartesian coordinates (in Å) and electronic energies (in Hartree) including zero-point energy as optimized at the noted level of theory

CO2 (B3LYP/aug-cc-pVDZ)
E=-188.602649
O 0.000000 0.000000 1.167362
C 0.000000 0.000000 0.000000
O 0.000000 0.000000 -1.167362

CO2 (B3LYP/aug-cc-pVTZ)
E= -188.651729
O 0.000000 -0.000000 1.160433
C 0.000000 0.000000 0.000000
O 0.000000 -0.000000 -1.160433

CO2 (BMK/aug-cc-pVDZ)
E=-188.520869
O 0.000000 0.000000 1.160433
C 0.000000 0.000000 0.000000
O 0.000000 0.000000 -1.160433

CO2 (CCSD/aug-cc-pVDZ)
E=-188.152791
O 0.000000 0.000000 1.170560
C 0.000000 0.000000 0.000000
O 0.000000 0.000000 -1.170560

CO2 (M06/aug-cc-pVDZ)
E=-188.516584
O 0.000000 0.000000 1.162135
C 0.000000 0.000000 0.000000
O 0.000000 0.000000 -1.162135

CO2 (M06L/aug-cc-pVDZ)
E=-188.592167
O 0.000000 0.000000 1.166462
C 0.000000 0.000000 0.000000
O 0.000000 0.000000 -1.166462

CO2 (MP2/aug-cc-pVDZ)
E=-188.158320
O 0.000000 0.000000 1.180225
C 0.000000 0.000000 0.000000
O 0.000000 0.000000 -1.180225

H2O (B3LYP/aug-cc-pVDZ)
E=-76.423405
H 0.000000 0.764616 -0.470433
O -0.000000 0.000000 0.117608
H -0.000000 -0.764616 -0.470433

H2O (B3LYP/aug-cc-pVTZ)
E= -76.444955
H 0.000000 -0.763466 -0.467930
O 0.000000 0.000000 0.116983
H 0.000000 0.763466 -0.467930

H2O (BMK/aug-cc-pVDZ)
E=-76.383180
H 0.000000 0.762316 -0.467227
O -0.000000 0.000000 0.116807
H -0.000000 -0.762316 -0.467227

H2O (CCSD/aug-cc-pVDZ)
E=-76.247202
H -0.000000 0.760745 -0.474237
O 0.000000 0.000000 0.118559
H -0.000000 -0.760745 -0.474237

H2O (M06/aug-cc-pVDZ)
E=-76.385179
H 0.000000 0.760939 -0.469513
O -0.000000 0.000000 0.117378
H -0.000000 -0.760939 -0.469513

H2O (M06L/aug-cc-pVDZ)
E=-76.409024
H -0.000000 0.755629 -0.475437
O 0.000000 0.000000 0.118859
H -0.000000 -0.755629 -0.475437

H2O (MP2/aug-cc-pVDZ)
E=-76.239579
H 0.000000 0.760498 -0.476423
O -0.000000 0.000000 0.119106
H -0.000000 -0.760498 -0.476423

$Mg(H_2O)CO_2^+$ Linear
(B3LYP/aug-cc-pVDZ)
E=-464.896640
C 1.790679 -0.121468 0.000003
O 0.606446 -0.065128 0.000001
O 2.938719 -0.176414 0.000004
Mg -1.312052 0.998904 -0.000011
O -2.278454 -0.831932 0.000006
H -1.891649 -1.721593 0.000015
H -3.241483 -0.948655 0.000006

$Mg(H_2O)CO_2^+$ Linear
(B3LYP/aug-cc-pVTZ)
E= -464.975215
C 1.789193 -0.119191 0.000003
O 0.611639 -0.051080 0.000001
O 2.929622 -0.185409 0.000004
Mg -1.306041 0.989745 -0.000011
O -2.280452 -0.827922 0.000006
H -1.905773 -1.720396 0.000014
H -3.243357 -0.926106 0.000006

$Mg(H_2O)CO_2^+$ Linear
(BMK/aug-cc-pVDZ)
E=-464.709629
C 1.757376 -0.099078 0.000003
O 0.581192 -0.023162 0.000001
O 2.897318 -0.176311 0.000005
Mg -1.355906 1.007943 -0.000010
O -2.162265 -0.885566 0.000006
H -1.696291 -1.731699 0.000015
H -3.107045 -1.088837 0.000005

$Mg(H_2O)CO_2^+$ Linear
(CCSD/aug-cc-pVDZ)
E= -463.829436
C 1.767065 -0.116832 0.000003
O 0.577034 -0.088424 0.000001
O 2.917567 -0.154177 0.000005
Mg -1.327715 1.023634 -0.000011
O -2.211505 -0.859978 0.000006
H -1.771687 -1.722252 0.000014
H -3.162894 -1.039721 0.000006

$Mg(H_2O)CO_2^+$ Linear
(M06/aug-cc-pVDZ)
E=-464.745138
C 1.775047 -0.112368 0.000003
O 0.597380 -0.045335 0.000001
O 2.918500 -0.176913 0.000005
Mg -1.337658 1.014042 -0.000011
O -2.221860 -0.867191 0.000006
H -1.778790 -1.727638 0.000014

H -3.171751 -1.051140 0.000007

$Mg(H_2O)CO_2^+$ Linear
(M06L/aug-cc-pVDZ)
E=-464.844642
C 1.759444 -0.108814 0.000003
O 0.578145 -0.052167 0.000001
O 2.908137 -0.165803 0.000005
Mg -1.340574 1.010014 -0.000012
O -2.186579 -0.866594 0.000006
H -1.735923 -1.721783 0.000013
H -3.131472 -1.068990 0.000007

$Mg(H_2O)CO_2^+$ Linear
(MP2/aug-cc-pVDZ)
E=-463.825792
C 1.767820 -0.113962 0.000003
O 0.571558 -0.096905 0.000002
O 2.930590 -0.142472 0.000005
Mg -1.349868 1.033312 -0.000011
O -2.194072 -0.874130 0.000006
H -1.731326 -1.726384 0.000014
H -3.141795 -1.081531 0.000007

$Mg(H_2O)_2CO_2^+$ Bidentate
(B3LYP/aug-cc-pVDZ)
E= -541.355710
O -1.383953 0.574093 0.957636
C -1.962428 -0.000378 0.000394
O -1.384419 -0.574742 -0.957266
Mg 0.301853 -0.000182 -0.000578
O 1.466357 -1.621047 0.244211
H 2.265047 -1.769717 0.771550
H 1.161725 -2.493362 -0.053241
O 1.464752 1.621993 -0.244189
H 2.264474 1.771083 -0.769837
H 1.159200 2.494080 0.052965

$Mg(H_2O)_2CO_2^+$ Bidentate
(B3LYP/aug-cc-pVTZ)
E= -541.455569
O 1.375886 0.538260 -0.972445
C 1.945867 -0.002100 0.000277
O 1.374984 -0.541326 0.972536
Mg -0.301956 0.000033 -0.001251
O -1.457405 -1.622052 -0.232845
H -2.231454 -1.775231 -0.790844
H -1.169362 -2.485251 0.098088
O -1.451565 1.625601 0.234018
H -2.228726 1.779950 0.787328
H -1.157386 2.488862 -0.091335

$Mg(H_2O)_2CO_2^+$ Bidentate
(BMK/aug-cc-pVDZ)
E= -541.131117
O -1.350296 0.566194 0.951489
C -1.930149 -0.000520 0.000353
O -1.350682 -0.567066 -0.951153
Mg 0.304089 -0.000153 -0.000446
O 1.430788 -1.619125 0.240656
H 2.216838 -1.788698 0.774918
H 1.116538 -2.477275 -0.077645
O 1.428849 1.620284 -0.240723
H 2.215857 1.790512 -0.773355
H 1.113324 2.478114 0.077165

$Mg(H_2O)_2CO_2^+$ Bidentate
(CCSD/aug-cc-pVDZ)
E= -540.116609
O -1.387399 0.635183 0.923308
C -1.971475 -0.000398 0.000192
O -1.387683 -0.635826 -0.923127
Mg 0.302063 -0.000089 -0.000111
O 1.472912 -1.626445 0.256344
H 2.268494 -1.774885 0.785577
H 1.158043 -2.501952 -0.015538
O 1.471524 1.627236 -0.256562
H 2.267760 1.776828 -0.784470
H 1.154971 2.502273 0.014904

$Mg(H_2O)_2CO_2^+$ Bidentate
(M06/aug-cc-pVDZ)
E= -541.170084
O -1.370131 0.661994 0.891249
C -1.948463 0.000270 0.000121
O -1.370626 -0.661641 -0.891178
Mg 0.309302 -0.000042 -0.000176
O 1.447199 -1.639507 0.260579
H 2.264360 -1.811490 0.746778
H 1.107719 -2.502449 -0.020185
O 1.447755 1.639128 -0.260502
H 2.264712 1.810919 -0.747111
H 1.108788 2.502122 0.020717

$Mg(H_2O)_2CO_2^+$ Bidentate
(M06L/aug-cc-pVDZ)
E= -541.296065
O -1.343471 0.728711 0.845039
C -1.923773 -0.000891 0.000139
O -1.343107 -0.730055 -0.844876
Mg 0.321946 -0.000041 -0.000128
O 1.415684 -1.655571 0.267388
H 2.234828 -1.859034 0.734880
H 1.039046 -2.508085 0.007768
O 1.413233 1.657129 -0.267486
H 2.231826 1.862509 -0.735100
H 1.034879 2.508738 -0.007365

$Mg(H_2O)_2CO_2^+$ Bidentate
(MP2/aug-cc-pVDZ)
E= -540.096936
O -1.390305 0.649550 0.921766
C -1.971765 -0.000464 0.000175
O -1.390457 -0.650296 -0.921591
Mg 0.305747 -0.000089 -0.000108
O 1.473825 -1.631793 0.259771
H 2.270562 -1.784730 0.789440
H 1.151055 -2.508989 -0.004306
O 1.472265 1.632706 -0.259967
H 2.269539 1.786832 -0.788468
H 1.147843 2.509407 0.003753

$Mg(H_2O)_2CO_2^+$ Monodentate
(B3LYP/aug-cc-pVDZ)
E= -541.345773
Mg 0.756293 0.053846 0.000035
O -0.556594 -1.319122 -0.000090
C -1.786901 -0.964814 -0.000044
O -2.319777 0.129701 0.000070
H -1.243327 1.357371 0.000037
O -0.282171 1.693741 0.000108
H -0.251775 2.658812 -0.000965
O 2.727683 -0.265219 -0.000003
H 3.123782 -1.152074 0.000184
H 3.464092 0.365815 -0.000102

$Mg(H_2O)_2CO_2^+$ Monodentate
(B3LYP/aug-cc-pVTZ)
E= -541.445105
Mg -0.757247 0.064435 0.003614
O 0.547565 -1.307280 0.003641
C 1.775802 -0.968247 -0.001687
O 2.317497 0.114266 -0.004975
H 1.246259 1.347145 -0.001668
O 0.289512 1.691486 0.001109
H 0.263720 2.654570 0.010594
O -2.719196 -0.269136 -0.003147
H -3.104959 -1.158045 0.008037
H -3.455902 0.357898 -0.023241

$Mg(H_2O)_2CO_2^+$ Monodentate
(BMK/aug-cc-pVDZ)
E= -541.117794
Mg 0.744215 0.120129 0.001094
O -0.481150 -1.297422 0.000874
C -1.725678 -1.018310 -0.000261
O -2.297220 0.048683 -0.001589
H -1.272129 1.363294 -0.000371
O -0.329836 1.719058 0.000299
H -0.325869 2.681435 0.003630
O 2.678033 -0.280809 -0.000798
H 3.010714 -1.190388 0.004767
H 3.452151 0.297892 -0.009866

$Mg(H_2O)_2CO_2^+$ Monodentate
(CCSD/aug-cc-pVDZ)
E= -540.102487
Mg 0.743904 0.064269 -0.001124
O -0.539700 -1.333623 -0.001001
C -1.786941 -0.987737 0.000381
O -2.313773 0.114109 0.001489
H -1.231563 1.415537 0.000660
O -0.280436 1.732639 0.000105
H -0.244021 2.695560 -0.005914
O 2.721328 -0.277741 0.000808
H 3.098275 -1.171229 -0.003119
H 3.472747 0.332264 0.008362

$Mg(H_2O)_2CO_2^+$ Monodentate
(M06/aug-cc-pVDZ)
E= -541.158630
Mg 0.742382 0.221600 0.000709
O -0.384541 -1.296391 0.001375
C -1.643267 -1.099431 -0.000130
O -2.292174 -0.074660 -0.001710
H -1.371995 1.333776 -0.000531
O -0.454806 1.750964 0.000116
H -0.520176 2.712267 0.004796
O 2.681335 -0.272622 -0.000742
H 2.942777 -1.206366 0.002772
H 3.501897 0.239377 -0.007077

$Mg(H_2O)_2CO_2^+$ Monodentate
(M06L/aug-cc-pVDZ)
E= -541.283210
Mg 0.765360 0.160750 0.000672
O -0.448026 -1.280146 0.000571
C -1.706765 -1.045986 -0.000232
O -2.331622 0.000561 -0.000963
H -1.328883 1.313452 -0.000383
O -0.395770 1.706612 0.000102
H -0.445974 2.667211 0.002976
O 2.712079 -0.269177 -0.000444
H 3.034698 -1.181805 0.003902
H 3.503145 0.285245 -0.007288

$Mg(H_2O)_2CO_2^+$ Monodentate
(MP2/aug-cc-pVDZ)
E= -540.083695
Mg 0.755532 0.081473 0.000362
O -0.524025 -1.333484 0.000187
C -1.775662 -0.997859 -0.000178
O -2.320272 0.104160 -0.000416
H -1.267753 1.346708 -0.000006
O -0.320462 1.712502 0.000326
H -0.325198 2.677979 -0.000523
O 2.736515 -0.260009 -0.000218
H 3.118390 -1.153465 0.001296
H 3.488112 0.352906 -0.003075

$Mg(H_2O)_2CO_2^+$ Linear
(B3LYP/aug-cc-pVDZ)
E= -541.352097
C 2.147570 -0.026045 -0.090556
O 0.976532 -0.191217 -0.107618
Mg -1.041845 0.050817 0.899728
O -1.668539 -1.514953 -0.366289
O 3.288109 0.135193 -0.068418
O -1.706489 1.540278 -0.416187
H -1.093000 -2.078577 -0.906224
H -1.888761 2.454039 -0.149134
H -2.040070 1.440911 -1.321458
H -2.478350 -2.024316 -0.208493

$Mg(H_2O)_2CO_2^+$ Linear
(B3LYP/aug-cc-pVTZ)
E= -541.452294
C 2.141352 -0.018524 -0.091054
O 0.973477 -0.160718 -0.116049
Mg -1.030965 0.045548 0.877115
O -1.660918 -1.529531 -0.353740
O 3.277533 0.120374 -0.060817
O -1.711334 1.541832 -0.403817
H -1.105149 -2.087474 -0.915721
H -1.910819 2.438476 -0.101333
H -2.024905 1.471742 -1.316657
H -2.465725 -2.033828 -0.169956

$Mg(H_2O)_2CO_2^+$ Linear
(BMK/aug-cc-pVDZ)
E= -541.124869
C 2.100101 -0.025307 -0.070339
O 0.936954 -0.195854 -0.055354
Mg -1.090208 0.052542 0.946756
O -1.591709 -1.462501 -0.410794
O 3.233414 0.139633 -0.086315
O -1.635628 1.488161 -0.456183
H -0.951797 -1.982389 -0.913077
H -1.833419 2.409952 -0.248900
H -1.891332 1.344544 -1.375776
H -2.385810 -2.006279 -0.332117

$Mg(H_2O)_2CO_2^+$ Linear
(CCSD/aug-cc-pVDZ)
E= -540.110071
C 2.115076 -0.022834 -0.090831
O 0.941257 -0.197116 -0.129270
Mg -1.041631 0.060465 0.952899
O -1.628651 -1.480399 -0.387126
O 3.256455 0.142846 -0.061141
O -1.678689 1.499489 -0.445907

H -1.005219 -2.020516 -0.893465
H -1.899065 2.416875 -0.232508
H -2.008176 1.339685 -1.341322
H -2.401403 -2.043185 -0.234950

$Mg(H_2O)_2CO_2^+$ Linear
(M06/aug-cc-pVDZ)
E= -541.165622
C 2.124907 -0.020844 -0.077905
O 0.959369 -0.182590 -0.060474
Mg -1.071003 0.053830 0.929629
O -1.618716 -1.505588 -0.390517
O 3.261756 0.137265 -0.090557
O -1.680900 1.516462 -0.443078
H -1.005560 -2.027195 -0.926716
H -1.879985 2.436203 -0.222452
H -1.964356 1.385660 -1.358732
H -2.419572 -2.039959 -0.303212

$Mg(H_2O)_2CO_2^+$ Linear
(M06L/aug-cc-pVDZ)
E= -541.289767
C 2.115277 -0.024707 -0.085772
O 0.945612 -0.180973 -0.077603
Mg -1.050340 0.057654 0.954131
O -1.633166 -1.458093 -0.403459
O 3.257857 0.127823 -0.089323
O -1.670181 1.480854 -0.447983
H -1.013373 -2.051647 -0.847444
H -1.881703 2.405851 -0.272434
H -1.945837 1.313903 -1.358719
H -2.447637 -1.968589 -0.309403

$Mg(H_2O)_2CO_2^+$ Linear
(MP2/aug-cc-pVDZ)
E= -540.098744
C 2.117788 -0.023918 -0.088180
O 0.938923 -0.208259 -0.128256
Mg -1.065878 0.065957 0.972261
O -1.617459 -1.472472 -0.397974
O 3.269653 0.151650 -0.058073
O -1.674967 1.489537 -0.460727
H -0.970918 -2.009038 -0.882351
H -1.892518 2.415085 -0.272633
H -1.996126 1.311816 -1.357654
H -2.385835 -2.049488 -0.265167

$Mg(H_2O)_2^+$(B3LYP/aug-cc-pVDZ)
E=-352.739150
mg -0.000000 -0.000000 0.932748
o 0.000000 1.526256 -0.495342
o -0.000000 -1.526256 -0.495342
h 0.328389 2.421762 -0.316572
h -0.513228 1.583005 -1.317179
h -0.328389 -2.421762 -0.316572
h 0.513228 -1.583005 -1.317179

$Mg(H_2O)_2^+$(B3LYP/aug-cc-pVDZ)
E= -352.790216
mg 0.000000 0.922135 0.000000
o 1.528020 -0.490259 0.000059
o -1.528020 -0.490259 -0.000059
h 2.413725 -0.310037 0.347088
h 1.594802 -1.300704 -0.525767
h -2.413725 -0.310037 -0.347088
h -1.594802 -1.300704 0.525768

$Mg(H_2O)_2^+$(BMK/aug-cc-pVDZ)
E=-352.594089
mg -0.000000 -0.000000 0.962176
o 0.000000 1.468096 -0.510501
o -0.000000 -1.468096 -0.510501
h 0.318107 2.368736 -0.361862
h -0.515950 1.493348 -1.327186
h -0.318107 -2.368736 -0.361862
h 0.515950 -1.493348 -1.327186

$Mg(H_2O)_2^+$(CCSD/aug-cc-pVDZ)
E= -351.944141
Mg 0.000000 0.000000 0.976575
O 0.000000 1.479059 -0.520927
O -0.000000 -1.479059 -0.520927
H 0.278744 2.393653 -0.364656
H -0.536073 1.500764 -1.327372
H -0.278744 -2.393653 -0.364656
H 0.536073 -1.500764 -1.327372

$Mg(H_2O)_2^+$(M06/aug-cc-pVDZ)
E=-352.635033
mg -0.000000 0.000000 0.955922
o 0.000000 1.500988 -0.505547
o -0.000000 -1.500988 -0.505547
h 0.318448 2.402390 -0.354287
h -0.495280 1.529914 -1.336866
h -0.318448 -2.402390 -0.354287
h 0.495280 -1.529914 -1.336866

$Mg(H_2O)_2^+$(M06L/aug-cc-pVDZ)
E=-352.682988
mg -0.000000 -0.000000 0.974756
o 0.000000 1.460269 -0.516689
o -0.000000 -1.460269 -0.516689
h 0.280091 2.375707 -0.383022
h -0.519890 1.469228 -1.331996
h -0.280091 -2.375707 -0.383022
h 0.519890 -1.469228 -1.331996

$Mg(H_2O)_2^+$(MP2/aug-cc-pVDZ)
E=-351.927885
mg 0.000000 0.000000 0.986387
o -0.000000 1.473161 -0.526834
o -0.000000 -1.473161 -0.526834
h 0.271773 2.392712 -0.375164
h -0.546627 1.492408 -1.328483
h -0.271773 -2.392712 -0.375164
h 0.546627 -1.492408 -1.328483

$Mg(H_2O)_3CO_2^+$ Bidentate
(B3LYP/aug-cc-pVDZ)
E= -617.821971
C -1.937338 0.662326 -0.000675
O -1.030414 1.532480 -0.001285
Mg 0.270037 -0.052420 -0.000018
O 0.572269 -1.157153 -1.684536
O -1.755085 -0.579969 0.000790
O 1.871217 1.224956 -0.001649
O 0.572999 -1.153137 1.687001
H 1.241167 -1.181001 -2.381409
H -0.202766 -1.639471 -2.011430
H -0.201654 -1.635171 2.015212
H 1.241858 -1.174474 2.383994
H 2.836546 1.185513 -0.002054
H 1.620536 2.162275 -0.002616

$Mg(H_2O)_3CO_2^+$ Bidentate
(B3LYP/aug-cc-pVTZ)
E= -617.943191
C -1.934766 0.641628 -0.000806
O -1.040239 1.514480 -0.001671
Mg 0.261385 -0.058020 0.000086
O 0.589393 -1.139525 -1.685470
O -1.751342 -0.593227 0.000528
O 1.846882 1.229780 -0.001905
O 0.589670 -1.134725 1.688650
H 1.266229 -1.132095 -2.372071
H -0.167451 -1.636172 -2.026003
H -0.166881 -1.630916 2.030492
H 1.266058 -1.124175 2.375655
H 2.810173 1.192968 -0.002296
H 1.588932 2.162606 -0.003025

$Mg(H_2O)_3CO_2^+$ Bidentate
(BMK/aug-cc-pVDZ)
E= -617.561026
C -1.874961 0.722783 0.000118
O -0.936081 1.546443 -0.000377
Mg 0.276745 -0.071272 -0.000055
O 0.501948 -1.153100 -1.675657
O -1.742050 -0.517711 0.000392
O 1.888257 1.147856 -0.000397
O 0.502540 -1.152267 1.676033
H 1.163067 -1.230250 -2.371515
H -0.303304 -1.588499 -1.986460
H -0.302575 -1.587597 1.987288
H 1.163776 -1.228773 2.371851
H 2.849903 1.100698 -0.000543
H 1.641046 2.083230 -0.000622

$Mg(H_2O)_3CO_2^+$ Bidentate
(CCSD/aug-cc-pVDZ)
E= -616.410182
C -1.933224 0.680698 -0.000662
O -1.012873 1.545450 -0.001945
Mg 0.270703 -0.056344 0.000025
O 0.557982 -1.164849 -1.691052
O -1.758993 -0.568816 0.000628
O 1.888894 1.207826 -0.001768
O 0.558423 -1.159961 1.694236
H 1.216307 -1.183845 -2.396194
H -0.228809 -1.618712 -2.027312
H -0.228131 -1.613197 2.031892
H 1.216555 -1.175999 2.399634
H 2.852382 1.158207 -0.002107
H 1.655132 2.148284 -0.003025

$Mg(H_2O)_3CO_2^+$ Bidentate
(M06/aug-cc-pVDZ)
E= -617.603171
C -1.906178 0.706931 -0.000655
O -0.980882 1.549165 -0.000098
Mg 0.277716 -0.054473 0.000038
O 0.526872 -1.172638 -1.686157
O -1.754927 -0.532898 -0.000171
O 1.900965 1.190599 -0.000004
O 0.526117 -1.172112 1.686705
H 1.189549 -1.254376 -2.381442
H -0.269618 -1.626662 -1.996363
H -0.270476 -1.626189 1.996570
H 1.188408 -1.253576 2.382388
H 2.863863 1.147777 0.000238
H 1.657588 2.128186 -0.000109

$Mg(H_2O)_3CO_2^+$ Bidentate
(M06L/aug-cc-pVDZ)
E= -617.752401
C -1.859997 0.765894 -0.003438
O -0.899331 1.576937 -0.007449
Mg 0.275502 -0.075134 0.000131
O 0.463763 -1.175817 -1.688490
O -1.757448 -0.485794 0.002972
O 1.939916 1.100469 -0.006406
O 0.465653 -1.157274 1.700338
H 1.102138 -1.250691 -2.405550
H -0.351474 -1.587746 -2.004426
H -0.348942 -1.566295 2.021650
H 1.104715 -1.223500 2.417637
H 2.899282 1.018082 -0.007011
H 1.747814 2.048232 -0.010962

$Mg(H_2O)_3CO_2^+$ Bidentate
(MP2/aug-cc-pVDZ)
E= -616.383242
C -1.912740 0.720883 -0.000443
O -0.973907 1.573186 -0.001399
Mg 0.280249 -0.062288 -0.000003
O 0.521188 -1.181233 -1.694900
O -1.769214 -0.537525 0.000586
O 1.920352 1.176620 -0.001453
O 0.521844 -1.177495 1.697276
H 1.169030 -1.221227 -2.411423
H -0.287370 -1.605354 -2.024192
H -0.286464 -1.601202 2.027708
H 1.169638 -1.215047 2.413978
H 2.885017 1.113872 -0.001858
H 1.701491 2.122694 -0.002402

$Mg(H_2O)_3CO_2^+$ Monodentate
(B3LYP/aug-cc-pVDZ)
E= -617.818549
C 2.022118 0.633562 -0.676961
O 0.792346 0.910141 -0.879269
Mg -0.605106 -0.028599 0.049418
O -1.730143 1.518657 0.716005
O 2.557237 -0.181600 0.060644
O -1.932496 -1.233114 -0.905236
O 0.497212 -1.209793 1.173258
H 0.496938 -1.770830 1.957322
H 1.454001 -0.971336 0.906037
H -2.586675 -0.975349 -1.571081
H -1.807970 -2.189442 -0.998819
H -2.515781 1.599615 1.274269
H -1.385202 2.414839 0.577809

$Mg(H_2O)_3CO_2^+$ Monodentate
(B3LYP/aug-cc-pVTZ)
E= -617.939853
C 2.015203 0.615283 -0.687788
O 0.790057 0.880422 -0.894838
Mg -0.607388 -0.028299 0.048347
O -1.722365 1.520867 0.702770
O 2.551513 -0.176145 0.063187
O -1.919578 -1.238918 -0.901669
O 0.489085 -1.176983 1.200390
H 0.485442 -1.713633 1.998911
H 1.443862 -0.947855 0.926623
H -2.561951 -0.982766 -1.576431
H -1.795161 -2.194145 -0.982280
H -2.501516 1.597485 1.267085
H -1.382933 2.414853 0.553935

$Mg(H_2O)_3CO_2^+$ Monodentate
(BMK/aug-cc-pVDZ)
E= -617.554048
C 1.998563 0.638633 -0.672907
O 0.769080 0.909881 -0.840900
Mg -0.608489 -0.040155 0.060641
O -1.668521 1.521918 0.719884
O 2.539484 -0.190228 0.030038
O -1.924601 -1.198221 -0.911550
O 0.475374 -1.231508 1.159057
H 0.464644 -1.817526 1.920492
H 1.425347 -1.010269 0.899906
H -2.575929 -0.913683 -1.564741
H -1.810683 -2.149692 -1.031678
H -2.441976 1.655303 1.279310
H -1.277456 2.391197 0.554227

$Mg(H_2O)_3CO_2^+$ Monodentate
(CCSD/aug-cc-pVDZ)
E= -616.402094
C 2.027710 0.665727 -0.653894
O 0.786000 0.959294 -0.828867
Mg -0.605889 -0.032117 0.054374
O -1.708105 1.535263 0.729941
O 2.563600 -0.196219 0.032228
O -1.954110 -1.216986 -0.911238
O 0.489527 -1.290148 1.129192
H 0.485734 -1.850468 1.912144
H 1.435801 -1.052264 0.885379
H -2.608742 -0.938811 -1.566640
H -1.815010 -2.163449 -1.057656
H -2.485539 1.651633 1.290646
H -1.323122 2.414768 0.596958

$Mg(H_2O)_3CO_2^+$ Monodentate
(M06/aug-cc-pVDZ)
E= -617.596426
C 1.985616 0.658316 -0.685146
O 0.756368 0.933596 -0.847629
Mg -0.601762 -0.045408 0.079610
O -1.710429 1.526067 0.719056
O 2.536752 -0.169373 0.015170
O -1.893911 -1.241146 -0.940288
O 0.498245 -1.233187 1.194988
H 0.496478 -1.819619 1.957631
H 1.446671 -1.013598 0.930378
H -2.534379 -0.969349 -1.610802
H -1.767668 -2.192135 -1.059324
H -2.489183 1.660007 1.272775
H -1.340666 2.402032 0.534524

$Mg(H_2O)_3CO_2^+$ Monodentate
(M06L/aug-cc-pVDZ)
E= -617.744001
C 1.997087 0.676583 -0.653079
O 0.757891 0.948126 -0.786599
Mg -0.620322 -0.048967 0.083841
O -1.679102 1.552467 0.697015
O 2.568715 -0.185572 -0.002681
O -1.954017 -1.221446 -0.881728
O 0.512118 -1.288192 1.098823
H 0.534404 -1.890989 1.846735
H 1.460311 -1.047552 0.837881
H -2.641926 -0.952284 -1.502576
H -1.822633 -2.165532 -1.035731
H -2.445601 1.732406 1.252588
H -1.268058 2.408994 0.514846

$Mg(H_2O)_3CO_2^+$ Monodentate
(MP2/aug-cc-pVDZ)
E= -616.375857
C 2.019030 0.673244 -0.656138
O 0.775002 0.967011 -0.827082
Mg -0.612819 -0.041621 0.066979
O -1.700211 1.548107 0.722449
O 2.563752 -0.197670 0.025552
O -1.970003 -1.226875 -0.890211
O 0.515491 -1.295179 1.107959
H 0.539783 -1.856026 1.892606
H 1.461361 -1.030223 0.843755
H -2.636752 -0.951004 -1.537006
H -1.827784 -2.173760 -1.043356
H -2.466864 1.686644 1.296081
H -1.302346 2.421215 0.571665

$Mg(H_2O)_3CO_2^+$ Linear
(B3LYP/aug-cc-pVDZ)
E= -617.803248
Mg -1.766151 -0.001740 0.846936
O -2.427114 1.691905 -0.241652
O -0.302219 -0.416275 -0.586337
O -3.179118 -1.252032 -0.123959
H -3.895073 -0.927360 -0.692591
H -3.494799 -2.080995 0.267483
H -0.394262 -1.102728 -1.263517
H 0.654907 -0.299364 -0.438488
H -2.912601 2.425560 0.165621
H -1.947461 2.064320 -0.998320
O 2.507371 -0.056284 -0.185738
C 3.662804 -0.011894 0.020023
O 4.801864 0.034287 0.222242

$Mg(H_2O)_3CO_2^+$ Linear
(B3LYP/aug-cc-pVTZ)
E= -617.924937
Mg -1.759245 0.007727 0.812293
O -2.382620 1.716873 -0.245514
O -0.302982 -0.458092 -0.593271
O -3.201699 -1.228536 -0.100411
H -3.912247 -0.924871 -0.683325
H -3.516634 -2.037278 0.326697
H -0.395950 -1.148543 -1.262844
H 0.650823 -0.342130 -0.436461
H -2.855354 2.443456 0.183415
H -1.927429 2.090566 -1.013575
O 2.497335 -0.118295 -0.129545
C 3.650196 -0.018647 0.029995
O 4.785785 0.080293 0.188567

$Mg(H_2O)_3CO_2^+$ Linear
(BMK/aug-cc-pVDZ)
E= -617.535316
Mg -1.732851 -0.001941 0.913947
O -2.504420 1.588493 -0.244953
O -0.330985 -0.299950 -0.588446
O -3.042867 -1.272867 -0.160806
H -3.751336 -0.919661 -0.713513
H -3.348732 -2.127496 0.167383
H -0.454692 -0.971783 -1.269114
H 0.627996 -0.197771 -0.467347
H -3.023464 2.317396 0.116895
H -2.001799 1.947214 -0.987341
O 2.486813 -0.037702 -0.219405
C 3.632070 -0.006931 0.007777
O 4.760687 0.024148 0.230987

$Mg(H_2O)_3CO_2^+$ Linear
(CCSD/aug-cc-pVDZ)
E= -616.385267
Mg -1.725009 -0.013422 0.909018
O -2.444490 1.637430 -0.241933
O -0.335474 -0.363016 -0.636976
O -3.120464 -1.247223 -0.141832
H -3.831831 -0.869214 -0.678809
H -3.478786 -2.063631 0.234753
H -0.459950 -1.071394 -1.283330
H 0.625290 -0.275308 -0.515144
H -2.890453 2.402506 0.148318
H -1.929892 1.980179 -0.987019
O 2.479896 -0.015000 -0.206994
C 3.636792 -0.008241 0.016775
O 4.776154 0.001231 0.236781

$Mg(H_2O)_3CO_2^+$ Linear
(M06/aug-cc-pVDZ)
E= -617.578435
Mg -1.812786 -0.001886 0.897147
O -2.400053 1.669953 -0.271958
O -0.296002 -0.402965 -0.490515
O -3.155683 -1.232512 -0.198977
H -3.806163 -0.884771 -0.825118
H -3.513317 -2.069874 0.125083
H -0.387043 -1.085955 -1.167475
H 0.660763 -0.286281 -0.349954
H -2.912773 2.422828 0.050616
H -1.866326 1.998462 -1.009040
O 2.504925 -0.077746 -0.163644
C 3.659857 -0.016161 0.005805
O 4.799205 0.046418 0.172005

$Mg(H_2O)_3CO_2^+$ Linear
(M06L/aug-cc-pVDZ)
E= -617.725568
Mg -1.737308 -0.014461 0.928613
O -2.475992 1.605600 -0.247515
O -0.328087 -0.325528 -0.584416
O -3.073351 -1.256219 -0.174624
H -3.778671 -0.875746 -0.714597
H -3.423047 -2.095755 0.149058
H -0.443260 -1.026215 -1.237944
H 0.632751 -0.229772 -0.471513
H -2.969478 2.357447 0.103071
H -1.946418 1.961124 -0.973580
O 2.485375 -0.014428 -0.239464
C 3.632188 -0.005829 0.003964
O 4.764893 0.005254 0.243314

$Mg(H_2O)_3CO_2^+$ Linear
(MP2/aug-cc-pVDZ)
E= -616.366729
Mg -1.717220 -0.020782 0.926828
O -2.438450 1.627689 -0.242407
O -0.335603 -0.354890 -0.641351
O -3.109532 -1.244409 -0.156239
H -3.816946 -0.848237 -0.688621
H -3.484200 -2.060879 0.209055
H -0.466340 -1.068996 -1.282893
H 0.629374 -0.274848 -0.523940
H -2.877844 2.403534 0.138692
H -1.907695 1.962704 -0.982059
O 2.456715 0.014696 -0.197432
C 3.623669 -0.005762 0.015797
O 4.775405 -0.021753 0.226561

$Mg(H_2O)_3^+$ (B3LYP/aug-cc-pVDZ)
E= -429.193068
H 2.279466 -1.308381 0.013546
H 1.166431 -1.713776 -0.991594
O 1.399948 -1.061903 -0.311969
O 0.219827 1.743079 -0.312002
H 0.901646 1.866774 -0.991100
H -0.007006 2.628019 0.013102
Mg 0.000017 0.000047 0.868464
O -1.619822 -0.681195 -0.311904
H -2.273109 -1.319584 0.013564
H -2.067261 -0.153465 -0.992087

$Mg(H_2O)_3^+$ (B3LYP/aug-cc-pVTZ)
E= -429.265766
H -2.446746 0.944707 0.048173
H -1.442979 1.518668 -0.989851
O -1.550177 0.846287 -0.301767
O 0.041067 -1.764979 -0.301264
H -0.596446 -2.007614 -0.988094
H 0.404909 -2.590973 0.047046
Mg 0.000441 0.000873 0.838894
O 1.508685 0.917769 -0.301993
H 2.042883 1.645658 0.045539
H 2.036488 0.486457 -0.989345

$Mg(H_2O)_3^+$ (BMK/aug-cc-pVDZ)
E= -429.007648
O -0.505109 1.611763 -0.332253
Mg 0.001121 -0.001444 0.929993
O 1.651834 -0.369891 -0.333335
O -1.148558 -1.240744 -0.332424
H -1.055197 2.353742 -0.050837
H 0.100144 1.955506 -1.001694
H -1.745362 -0.886938 -1.004201
H -1.515945 -2.090042 -0.056992
H 2.569173 -0.266916 -0.050322
H 1.648398 -1.057050 -1.011764

$Mg(H_2O)_3^+$ (CCSD/aug-cc-pVDZ)
E= -428.223419
H 2.519467 -0.654523 -0.037053
H 1.498410 -1.307264 -0.994621
O 1.601514 -0.601465 -0.339833
O -0.279819 1.687656 -0.339789
H 0.383271 1.951397 -0.994223
H -0.693042 2.509093 -0.037106
Mg -0.000006 -0.000085 0.937538
O -1.321698 -1.086097 -0.339846
H -1.826564 -1.854632 -0.037198
H -1.881446 -0.643794 -0.994507

$Mg(H_2O)_3^+$ (M06/aug-cc-pVDZ)
E= -429.054063
H 2.490433 -0.816415 -0.038775
H 1.444995 -1.410235 -1.012331
O 1.567865 -0.746505 -0.319054
O -0.134527 1.728242 -0.317864
H 0.507115 1.954386 -1.005999
H -0.538067 2.561667 -0.039960
Mg -0.000705 -0.002317 0.900408
O -1.432791 -0.979510 -0.319526
H -1.957466 -1.743465 -0.044424
H -1.942933 -0.535947 -1.011855

$Mg(H_2O)_3^+$ (M06L/aug-cc-pVDZ)
E=-429.125948

Mg -0.000045 0.000468 0.949061
O -0.513916 1.610236 -0.340841
O -1.138489 -1.250181 -0.340698
O 1.652477 -0.360545 -0.340523
H -0.507816 2.539686 -0.0791
H -1.22465 1.532313 -0.991181
H -1.94555 -1.710835 -0.078299
H -0.716564 -1.825196 -0.993004
H 2.454322 -0.830455 -0.078564
H 1.94022 0.292784 -0.992079

$Mg(H_2O)_3^+$ (MP2/aug-cc-pVDZ)

E= -428.199279
H 2.465123 -0.835155 -0.053040
H 1.383090 -1.407328 -0.995954
O 1.549610 -0.706168 -0.346505
O -0.163185 1.695050 -0.346462
H 0.527523 1.901470 -0.995656
H -0.509355 2.552379 -0.053071
Mg -0.000011 -0.000075 0.955219
O -1.386423 -0.988798 -0.346516
H -1.955858 -1.717234 -0.053174
H -1.910405 -0.493899 -0.995866

Cartesian coordinates (in Å) and electronic energies (in Hartree) including zero-point energy as optimized at the M06L/aug-cc-pVDZ level of theory

Ia
E=-464.844642
C 1.759444 -0.108814 0.000003
O 0.578145 -0.052167 0.000001
O 2.908137 -0.165803 0.000005
Mg -1.340574 1.010014 -0.000012
O -2.186579 -0.866594 0.000006
H -1.735923 -1.721783 0.000013
H -3.131472 -1.068990 0.000007

Mg(H2O)+
E=-276.233785
Mg 0.985283 -0.000013 -0.000008
O -1.064836 -0.000046 0.000008
H -1.652756 -0.769204 0.000017
H -1.651953 0.769726 0.000009

IIb
E= -541.296065
O -1.343471 0.728711 0.845039
C -1.923773 -0.000891 0.000139
O -1.343107 -0.730055 -0.844876
Mg 0.321946 -0.000041 -0.000128
O 1.415684 -1.655571 0.267388
H 2.234828 -1.859034 0.734880
H 1.039046 -2.508085 0.007768
O 1.413233 1.657129 -0.267486
H 2.231826 1.862509 -0.735100
H 1.034879 2.508738 -0.007365

IIc
E= -541.283210
Mg 0.765360 0.160750 0.000672
O -0.448026 -1.280146 0.000571
C -1.706765 -1.045986 -0.000232
O -2.331622 0.000561 -0.000963
H -1.328883 1.313452 -0.000383
O -0.395770 1.706612 0.000102
H -0.445974 2.667211 0.002976
O 2.712079 -0.269177 -0.000444
H 3.034698 -1.181805 0.003902
H 3.503145 0.285245 -0.007288

IIa
E= -541.289767
C 2.115278 -0.024708 -0.085772
O 0.945613 -0.180973 -0.077603
Mg -1.050341 0.057653 0.954128
O -1.633170 -1.458095 -0.403458
O 3.257859 0.127821 -0.089322
O -1.670179 1.480858 -0.447981
H -1.013379 -2.051649 -0.847447
H -1.881699 2.405854 -0.272427
H -1.945841 1.313912 -1.358717
H -2.447640 -1.968591 -0.309399

Mg(H2O)2+
E=-352.682988
mg 0.000000 0.000000 0.974756
o 0.000000 1.460269 -0.516689
o -0.000000 -1.460269 -0.516689
h 0.280091 2.375707 -0.383022
h -0.519890 1.469228 -1.331996
h -0.280091 -2.375707 -0.383022
h 0.519890 -1.469228 -1.331996

IIIb
E= -617.752401
C -1.859997 0.765894 -0.003438
O -0.899331 1.576937 -0.007449
Mg 0.275502 -0.075134 0.000131
O 0.463763 -1.175817 -1.688490
O -1.757448 -0.485794 0.002972
O 1.939916 1.100469 -0.006406
O 0.465653 -1.157274 1.700338
H 1.102138 -1.250691 -2.405550
H -0.351474 -1.587746 -2.004426
H -0.348942 -1.566295 2.021650
H 1.104715 -1.223500 2.417637
H 2.899282 1.018082 -0.007011
H 1.747814 2.048232 -0.010962

IIIc
E= -617.744001
C 1.997087 0.676583 -0.653079
O 0.757891 0.948126 -0.786599
Mg -0.620322 -0.048967 0.083841
O -1.679102 1.552467 0.697015
O 2.568715 -0.185572 -0.002681
O -1.954017 -1.221446 -0.881728

O 0.512118 -1.288192 1.098823
H 0.534404 -1.890989 1.846735
H 1.460311 -1.047552 0.837881
H -2.641926 -0.952284 -1.502576
H -1.822633 -2.165532 -1.035731
H -2.445601 1.732406 1.252588
H -1.268058 2.408994 0.514846

IIIa
E= -617.725568
Mg -1.737308 -0.014462 0.928613
O -2.475992 1.605600 -0.247515
O -0.328087 -0.325528 -0.584416
O -3.073351 -1.256219 -0.174624
H -3.778671 -0.875746 -0.714597
H -3.423047 -2.095755 0.149058
H -0.443260 -1.026215 -1.237944
H 0.632751 -0.229772 -0.471514
H -2.969478 2.357447 0.103071
H -1.946418 1.961124 -0.973580
O 2.485375 -0.014428 -0.239464
C 3.632188 -0.005829 0.003964
O 4.764893 0.005254 0.243314

Mg(H2O)3+
E=-429.125948
Mg -0.000045 0.000468 0.949061
O -0.513916 1.610236 -0.340841
O -1.138489 -1.250181 -0.340698
O 1.652477 -0.360545 -0.340523
H -0.507816 2.539686 -0.0791
H -1.22465 1.532313 -0.991181
H -1.94555 -1.710835 -0.078299
H -0.716564 -1.825196 -0.993004
H 2.454322 -0.830455 -0.078564
H 1.94022 0.292784 -0.992079

IVa
E= -694.158428
C -3.922184 -0.000093 0.029239
O -5.056576 -0.000166 -0.204490
O -2.774308 -0.000043 0.266471
O 0.071757 0.000707 0.692929
Mg 1.639705 -0.000213 -0.713692
O 3.004215 0.000155 0.909727
O 1.815442 2.167632 -0.423898
H 3.546957 0.772492 1.107811
H 3.547946 -0.771453 1.107919
H 0.192936 0.000088 1.649579
H -0.885710 0.000339 0.540669
H 2.118790 2.638630 -1.211909
H 1.103139 2.714734 -0.065997
O 1.815625 -2.167918 -0.423563
H 1.104681 -2.716131 -0.064653
H 2.118652 -2.638503 -1.211953

Mg(H2O)4+
E=-505.559786
O 0.001404 1.627089 0.648352
Mg -0.000304 -0.016168 -0.706479
O -2.168856 -0.184253 -0.410530
O -0.000280 -1.291921 0.985604
H 0.000870 1.562471 1.612048
H 0.000758 2.568923 0.442423
H -0.772255 -1.821165 1.218984
H 0.771892 -1.820876 1.218995
H -2.736561 0.522654 -0.074411
H -2.637210 -0.531252 -1.182105
O 2.168238 -0.185420 -0.410930
H 2.636899 -0.533497 -1.181821
H 2.735211 0.522795 -0.076339

IVc
E= -694.191410
C 2.329920 -0.571595 -0.008551
O 1.139373 -1.022772 -0.014792
O 2.756632 0.574527 0.014701
O 0.461516 1.755217 0.050659
H 1.448039 1.536303 0.042823
H 0.369300 2.709804 0.084024
H -0.265157 -1.686499 1.881282
O -0.984979 -1.079633 1.661122
H -1.561148 -1.039778 2.430236
H -0.240768 -1.553819 -1.992978
O -0.959198 -0.961610 -1.732961
H -1.535221 -0.869677 -2.497709
Mg -0.525136 0.031125 0.004623
O -2.415065 0.932318 0.020970
H -3.275343 0.498487 0.021207
H -2.603816 1.876877 0.029344

IVb
E= -694.196131
C -2.120555 -0.000016 -0.121392
O -1.615178 -0.000052 1.024526
Mg 0.211430 -0.000002 0.005275
O 0.254206 -2.089413 -0.299472
O -1.490363 0.000031 -1.210315
O 0.254147 2.089442 -0.299315
O 1.951600 0.000082 -1.105207
O 1.080342 -0.000083 1.875325
H 2.297386 -0.771817 -1.566108
H 2.297337 0.772038 -1.566049
H 0.311066 2.757477 0.393032
H -0.473352 2.374788 -0.868436
H 0.311143 -2.757486 0.392836
H -0.473288 -2.374743 -0.868608
H 0.468014 -0.000114 2.621705
H 1.969832 -0.000080 2.242336

Mg(H2O)5+
E=-581.992195
O 0.006672 -0.004097 1.660110
Mg -0.000554 0.001227 -0.460688
O -1.908961 0.979589 -0.417290
O 1.906029 -0.980128 -0.423728
O 1.037281 1.874608 -0.088544
H 0.364500 0.682209 2.232643
H -0.366361 -0.684232 2.230307
H 1.953076 1.866238 -0.398850
H 0.644073 2.640520 -0.528592
H 2.498387 -1.419934 0.203889
H 2.233817 -1.187391 -1.311559
H -2.230367 1.185079 -1.308183
H -2.503468 1.423923 0.205172
O -1.039436 -1.872129 -0.089180
H -0.646384 -2.641889 -0.522455
H -1.953320 -1.861989 -0.405067

Vd
E= -770.609206
C -2.903594 -0.105024 -0.157740
O -2.459868 -1.024167 -0.848807
O -2.339332 0.763605 0.560431
O 0.064456 -1.024661 -1.256158
Mg 0.926588 0.114973 0.118737
O 2.262635 -1.471288 0.543907

O 2.593381 1.046761 -0.637303
O -0.077232 1.857652 -0.176764
O -0.215851 -0.232327 1.725809
H 0.050290 2.752939 0.150526
H -1.040852 1.642580 -0.011872
H -1.138164 0.095918 1.516928
H -0.304337 -0.965369 2.340441
H 2.008512 -2.308382 0.134919
H 2.793769 -1.707434 1.312290
H 3.467649 0.661856 -0.760293
H 2.593533 1.881703 -1.118221
H -0.953423 -1.093362 -1.124045
H 0.200010 -1.034571 -2.208011

Vb
E= -770.629497
C -0.100384 2.097192 0.128046
O 0.677064 1.451446 -0.625319
Mg -0.450263 -0.244021 0.039972
O -2.064547 -1.207134 0.912197
O -1.047655 1.623466 0.790609
O -1.563111 -0.033619 -1.744519
O 2.882883 -0.182882 -0.146599
O 0.710573 -1.660940 -0.880094
H 0.560481 -1.977487 -1.775978
H 1.653629 -1.403742 -0.828732
H 3.843204 -0.168135 -0.215685
H 2.573265 0.681669 -0.463667
H -2.520520 -0.110737 -1.818165
H -1.322366 0.727504 -2.287101
H -2.655236 -0.670249 1.454514
H -2.114759 -2.099637 1.271581
O 0.839214 -0.519736 1.645841
H 0.742386 0.027902 2.432133
H 1.770007 -0.426787 1.366226

Vc
E= -770.632528
C -2.372961 0.780403 -0.023598
O -1.154367 1.128718 -0.031255
Mg 0.473882 -0.026724 0.004122
O -0.704987 -1.696377 0.041751
O -2.897990 -0.327889 0.004997
O 0.639322 -0.220898 -2.109588
O 0.628987 -0.111451 2.121238
O 2.334856 -0.969435 0.026015
H -0.668093 -2.654902 0.068629
H -1.668921 -1.408807 0.033323
H 2.801530 -1.317905 -0.740154
H 2.803253 -1.286353 0.804731
H -0.036432 -0.784165 -2.506325
H 0.642656 0.576689 -2.651417
H 0.637416 0.712726 2.621651
H -0.053240 -0.647982 2.543259
O 1.319499 1.890130 -0.047716
H 0.611675 2.548629 -0.066593
H 2.158776 2.357958 -0.058514

Vid
E= -847.051510
C -3.168952 -0.065630 0.038707
O -2.479830 -1.121138 0.010117
O -2.853691 1.124902 -0.024969
O -0.362106 1.597130 0.453983
Mg 0.830789 0.015573 0.010787
O 2.178343 0.307777 1.614013
O -0.174330 -1.264043 1.276745
O -0.389068 -0.609222 -1.517307
O 2.172486 -1.434590 -0.759248
O 1.922604 1.438863 -1.094846
H 0.028373 -2.173487 1.513169
H -1.133582 -1.272176 0.996203
H -1.261818 -0.887554 -1.111280
H -0.619140 -0.130205 -2.319093
H 1.824402 -2.052499 -1.412357
H 2.941837 -1.858391 -0.366499
H 1.504090 2.297048 -1.229526
H 2.560271 1.327427 -1.806897
H 2.862421 0.981000 1.689440
H 1.927767 0.078065 2.515722
H -1.348831 1.473572 0.232647
H -0.356823 2.106675 1.268886

VIb
E= -847.064198
c -0.000153 -2.046711 -0.000096
o 0.927248 -1.479132 -0.623974
mg 0.000003 0.369304 -0.000033
o 1.244977 0.392027 1.663073
o -0.927499 -1.479066 0.623792
o -1.420827 1.552063 0.872204
o -1.245023 0.392320 -1.663073
o 1.420990 1.551990 -0.872116
o 3.316115 -0.212652 -0.068449
o -3.315957 -0.212592 0.068597
h 1.054711 -0.175819 2.417171
h 2.161971 0.185481 1.398073
h -2.317857 1.166950 0.838878
h -1.369589 2.117935 1.646690
h 1.369865 2.118119 -1.646421
h 2.318029 1.166893 -0.838784
h -2.161977 0.185680 -1.398035
h -1.054784 -0.175381 -2.417285
h -2.898499 -1.020913 0.408349
h -4.265557 -0.369477 0.092146
h 4.265712 -0.369564 -0.091934
h 2.898662 -1.020941 -0.408299

VIc
E=are -847.067019
C 1.464559 -1.805860 0.471649
O 2.285450 -1.310057 -0.306386
O 0.250097 -1.540460 0.673476
Mg -0.752662 0.080413 0.020891
O -1.976180 -0.508584 1.610188
O 0.547558 0.270818 -1.597622
O -1.946241 -0.996284 -1.363174
O 0.334185 1.491536 1.086568
O -1.987295 1.692068 -0.479882
H 0.936419 1.093683 -1.912100
H 1.323911 -0.298740 -1.356101
H -2.691690 1.708618 -1.134974
H -1.804029 2.606106 -0.241078
H -1.468884 -1.188142 -2.179915
H -2.353160 -1.831230 -1.106336
H 0.336767 1.500214 2.048080
H 1.288140 1.548847 0.817892
H -1.604710 -1.270641 2.073094
H -2.772326 -0.243048 2.077815
H 3.024271 0.448347 0.031770
O 2.795784 1.393713 0.063075
H 3.603020 1.854180 0.311337

Mg(H2O)6+
E= -658.427397
O 1.178255 0.791176 -1.511359

Mg -0.006344 0.000368 0.001123
O -1.242475 -0.480896 1.634173
O -1.257814 1.645629 -0.403099
O 1.201381 -1.695421 0.065149
O 1.189713 0.919373 1.431776
O -1.253597 -1.182449 -1.216220
H 2.126930 0.683595 1.141870
H 1.223278 1.858425 1.663936
H -1.947569 -0.910976 -1.827730
H -0.890065 -2.007117 -1.562237
H 1.248331 -2.364984 0.762465
H 2.135345 -1.319657 -0.004240
H 1.217015 0.519023 -2.439620
H 2.117170 0.669458 -1.160182
H -1.954988 2.040205 0.132989
H -0.896345 2.354701 -0.949515
H -1.938410 -1.144415 1.701343
H -0.888265 -0.361969 2.524090

VIId
E= -923.485710
Mg 1.025981 -0.086070 0.045821
O 0.596220 -2.113617 0.051629
H 0.931185 -2.812170 -0.517999
H -0.391641 -2.172616 -0.014966
O -0.336849 0.182783 -1.475111
H -1.015105 -0.526344 -1.421901
H -0.842455 1.014290 -1.400366
O 2.411492 -0.157458 -1.543445
H 2.063609 -0.143025 -2.442114
H 3.345358 -0.376761 -1.609715
O 1.212872 1.994678 0.211223
H 0.304847 2.359110 0.262679
H 1.683146 2.561039 -0.407990
O 2.637757 -0.371879 1.363103
H 3.021587 0.329822 1.899934
H 2.789100 -1.191147 1.846541
O -0.334789 0.131369 1.548052
H -1.310513 0.096840 1.315564
H -0.265204 -0.068976 2.484039
O -1.527722 2.389969 -0.293412
H -2.171608 1.779924 0.118720
H -2.034935 3.174159 -0.526802
O -2.772457 0.068222 0.637072
C -2.881934 -0.993815 -0.011534
O -2.027466 -1.752618 -0.507396

VIIb
E= -923.485405
C 1.742793 -1.922537 -0.205434
O 1.826144 -0.843284 0.441354
Mg -0.295380 -0.707130 0.043693
O -0.821026 0.909029 1.155379
O 0.705314 -2.420837 -0.685081
O 0.204657 0.529416 -1.589335
O -2.145087 -0.877458 -0.847994
O -0.811273 -1.938085 1.662833
O 1.099544 2.740907 1.141131
O 2.674807 1.429722 -0.762097
H -0.116799 1.598633 1.268979
H -1.592434 1.388796 0.815244
H 1.518492 3.156445 1.899814
H 1.825075 2.474722 0.548996
H -0.890562 -1.586104 2.555837
H -0.388118 -2.799857 1.748976
H -2.723450 -1.644564 -0.818756
H -2.711702 -0.083565 -0.844885
H 0.158484 0.148620 -2.472460
H 1.096628 0.933919 -1.520251
H 2.821989 0.585192 -0.296584
H 3.503289 1.647262 -1.200607
H -2.827505 2.329353 -1.166220
O -3.065016 1.704166 -0.471357
H -3.930098 2.003325 -0.168449

VIIc
E= -923.498397
C 1.615173 -0.000177 1.691100
O 2.510147 0.000000 0.835967
O 0.366349 -0.000094 1.582606
Mg -0.910134 -0.000029 0.015381
O -0.856403 -2.089043 -0.184124
O 0.380815 0.000154 -1.612516
O -0.856652 2.089153 -0.184099
O -2.261148 -0.000220 1.615095
O -2.595777 -0.000035 -1.258537
O 1.814631 2.176165 -0.748299
O 1.814814 -2.175916 -0.748705
H -1.213910 2.659536 0.503184
H 0.072109 2.382031 -0.312738
H -1.846238 -0.000272 2.485946
H -3.213626 -0.000302 1.741714
H 0.975845 -0.770922 -1.653779
H 0.975749 0.771310 -1.653688
H -1.213642 -2.659852 0.502816
H 0.072327 -2.381901 -0.313023
H -2.699725 0.765691 -1.834962
H -2.699600 -0.765653 -1.835127
H 2.439961 2.855017 -1.019988
H 2.303385 1.574551 -0.149852
H 2.303602 -1.574429 -0.150164
H 2.440130 -2.854700 -1.020597

Mg(H2O)7+
E= -734.854850
O -0.789792 1.505660 -0.685315
Mg 0.439239 -0.033394 -0.010552
O -0.356992 -0.295290 1.897537
O 1.594674 0.100646 -1.774751
O 1.816984 -1.514870 0.543161
O -0.915434 -1.343265 -0.886084
O 1.575562 1.559035 0.754556
H -0.710899 0.612551 2.148066
H -1.104986 -0.888349 2.073918
H 1.211997 -0.140346 -2.626789
H 1.961899 0.984570 -1.906872
H 2.309474 1.467989 1.376449
H 0.944450 2.181092 1.194093
H -1.623381 1.307060 -1.140516
H -1.080311 2.008936 0.128616
H -0.896595 -2.303607 -0.940251
H -1.837437 -1.085741 -0.653824
H 2.607644 -1.810196 0.078364
H 1.869557 -1.868305 1.438089
O -3.154204 -0.058895 -0.060219
H -4.109585 -0.195361 -0.073575
H -2.979063 0.506257 0.719774

VIIId
E= -999.912969
C -3.221175 -1.276311 -0.246693
O -2.238616 -1.974240 -0.560773
O -3.304614 -0.164602 0.317266
O -1.014790 0.231591 1.511985
Mg 0.567912 0.059513 0.219657
O 1.933461 0.097212 1.819351
O 0.316676 -1.998759 0.351126
O -0.631270 0.074199 -1.465866

O 2.163349 0.025216 -1.082182
O 0.488203 2.163639 0.240921
O -2.194298 2.213218 -0.626040
H 0.786586 -2.675464 -0.144462
H -0.643508 -2.162570 0.167502
H -1.234601 -0.699429 -1.437616
H -1.224524 0.846452 -1.518620
H 1.989689 -0.053126 -2.024354
H 3.127741 -0.104088 -0.949249
H -0.452625 2.419802 0.151479
H 0.963714 2.708798 -0.393044
H 2.173153 0.888914 2.311109
H 2.231408 -0.650491 2.346263
H -1.940758 0.072445 1.165061
H -1.034647 0.036391 2.451520
H -2.815532 1.556203 -0.252616
H -2.749999 2.914136 -0.981156
O 4.806427 -0.338498 -0.575946
H 5.456682 0.361138 -0.696530
H 5.283097 -1.147206 -0.789759

VIIIb
E= -999.912035
C 2.524366 0.883027 -0.891300
O 2.432185 -0.261007 -1.375468
Mg 0.860047 -0.564871 0.027074
O 2.168113 -1.069245 1.595450
O 1.856010 1.337728 0.077581
O -0.599547 -0.266595 1.410437
O -0.485336 0.031636 -1.437342
O 0.428304 -2.537200 -0.264315
O -3.113782 0.060299 0.486474
O -1.962386 2.206018 -0.793066
O -0.077268 3.175947 0.828273
H -0.420124 0.026146 2.307304
H -1.558958 -0.133121 1.243937
H -3.919288 0.170567 1.001099
H -2.978037 0.908066 0.017425
H 2.391163 -1.980097 1.814990
H 2.975970 -0.558299 1.717863
H 0.824632 -3.069483 -0.959486
H -0.546761 -2.703259 -0.299515
H -0.140559 0.018657 -2.335981
H -1.000771 0.866626 -1.355323
H -1.334843 2.709710 -0.213386
H -2.323036 2.847622 -1.412096
H 0.770910 2.773776 0.569050
H 0.124740 4.092069 1.037405
H -2.689913 -1.754011 -0.189822
O -2.241729 -2.614257 -0.199144
H -2.878380 -3.241268 -0.551576

VIIIc
E= -999.925177
C -1.258121 0.662098 1.529029
O -1.987129 1.340460 0.799252
O -0.022406 0.448929 1.516682
Mg 1.211622 -0.215219 0.072945
O 2.471466 -0.434185 1.733555
O 2.818381 -0.938410 -1.100789
O 1.928877 1.693094 -0.441335
O -0.057682 -0.013020 -1.538432
O 0.529020 -2.190238 0.161922
O -1.931650 -1.920036 -0.931752
O -0.515543 2.651660 -1.075712
O -3.948840 -0.558474 0.150583
H 0.589318 -2.676173 0.989642
H -0.408846 -2.276777 -0.128669
H 2.087775 -0.180261 2.580728
H 3.376415 -0.714548 1.893847
H -0.381319 0.890949 -1.703819
H -0.847777 -0.591328 -1.527897
H 2.465938 2.198934 0.175422
H 1.160944 2.270829 -0.656654
H 2.718212 -1.799183 -1.521988
H 3.138683 -0.347678 -1.791820
H -2.303881 -2.541907 -1.564186
H -2.716347 -1.514964 -0.474788
H -1.144000 2.385688 -0.363467
H -0.866617 3.457775 -1.465319
H -4.602292 -0.830885 0.800435
H -3.532882 0.241333 0.517236

Mg(H2O)8+
E= -811.278419
O -1.716740 -1.374073 1.336950
Mg -0.793889 -0.093129 -0.003068
O 0.483304 -1.371423 -0.969120
O -1.979117 1.495736 0.731847
O 0.078986 1.564155 -1.138196
O -2.263444 -0.499492 -1.404749
O 0.649361 0.324240 1.423762
O 2.962709 -1.452968 0.015115
H 1.413886 -1.547636 -0.687270
H 0.217671 -2.071054 -1.571695
H -2.920137 1.138762 0.690523
H -1.869619 1.800106 1.645050
H 1.529014 0.683338 1.195134
H 0.769677 -0.260793 2.178810
H -2.690883 -1.138415 1.282862
H -1.685613 -2.340443 1.340062
H -2.474317 -0.205289 -2.301622
H -3.132794 -0.471523 -0.888653
H -0.520237 2.299834 -0.931194
H 0.054724 1.483778 -2.101447
H 3.702117 -2.039276 -0.170964
H 3.302983 -0.551934 -0.092735
H 2.212382 1.783987 -0.554907
O 2.807498 1.375033 0.089142
H 3.437360 2.064441 0.326862

TS(IIa/IIc)
E= -541.274069
C -1.579428 -0.886400 -0.079379
Mg 0.925287 0.230916 0.715805
O -2.354154 -0.071541 -0.447009
O -0.506998 -1.190547 0.421983
O 2.333691 -0.468181 -0.598399
O -0.209887 1.711230 -0.101905
H 3.237778 -0.170998 -0.763291
H 2.258376 -1.329400 -1.033018
H -0.079989 2.664588 -0.179306
H -1.144256 1.535540 -0.335139

TS(IIb/IIc)
E= -541.282663
c 1.697022 -1.040539 0.000057
o 0.474978 -1.423909 -0.000138
mg -0.637005 0.110761 -0.000172
o 0.238990 1.851898 -0.000013
o -2.584110 -0.323421 0.000140
o 2.170748 0.084271 0.000173
h 0.127572 2.808331 -0.000136
h 1.202797 1.620470 0.000072
h -3.384555 0.216730 0.000227
h -2.888736 -1.242131 0.000265

CO2
E=-188.592165 in
O 0.000000 0.000000 1.166402
C 0.000000 0.000000 0.000000
O 0.000000 0.000000 -1.166402

H2O
E=-76.409024
H 0.000000 0.755629 -0.475437
O 0.000000 0.000000 0.118859
H -0.000000 -0.755629 -0.475437

Mg(CO2)+
E=-388.392306
C -1.158468 -0.076675 0.000000
O -2.279108 -0.321234 0.000000
O -0.000000 0.170727 -0.000000
Mg 2.098639 0.138675 -0.000000

Mg
E= -199.775896
Mg 0.000000 0.000000 0.000000
